# Inferring Regulatory Networks from Experimental Morphological Phenotypes: A Computational Method Reverse-Engineers Planarian Regeneration

**DOI:** 10.1371/journal.pcbi.1004295

**Published:** 2015-06-04

**Authors:** Daniel Lobo, Michael Levin

**Affiliations:** Center for Regenerative and Developmental Biology, Department of Biology, Tufts University, Medford, Massachusetts, United States of America; University of Virginia, UNITED STATES

## Abstract

Transformative applications in biomedicine require the discovery of complex regulatory networks that explain the development and regeneration of anatomical structures, and reveal what external signals will trigger desired changes of large-scale pattern. Despite recent advances in bioinformatics, extracting mechanistic pathway models from experimental morphological data is a key open challenge that has resisted automation. The fundamental difficulty of manually predicting emergent behavior of even simple networks has limited the models invented by human scientists to pathway diagrams that show necessary subunit interactions but do not reveal the dynamics that are sufficient for complex, self-regulating pattern to emerge. To finally bridge the gap between high-resolution genetic data and the ability to understand and control patterning, it is critical to develop computational tools to efficiently extract regulatory pathways from the resultant experimental shape phenotypes. For example, planarian regeneration has been studied for over a century, but despite increasing insight into the pathways that control its stem cells, no constructive, mechanistic model has yet been found by human scientists that explains more than one or two key features of its remarkable ability to regenerate its correct anatomical pattern after drastic perturbations. We present a method to infer the molecular products, topology, and spatial and temporal non-linear dynamics of regulatory networks recapitulating *in silico* the rich dataset of morphological phenotypes resulting from genetic, surgical, and pharmacological experiments. We demonstrated our approach by inferring complete regulatory networks explaining the outcomes of the main functional regeneration experiments in the planarian literature; By analyzing all the datasets together, our system inferred the first systems-biology comprehensive dynamical model explaining patterning in planarian regeneration. This method provides an automated, highly generalizable framework for identifying the underlying control mechanisms responsible for the dynamic regulation of growth and form.

## Author Summary

Developmental and regenerative biology experiments are producing a huge number of morphological phenotypes from functional perturbation experiments. However, existing pathway models do not generally explain the dynamic regulation of anatomical shape due to the difficulty of inferring and testing non-linear regulatory networks responsible for appropriate form, shape, and pattern. We present a method that automates the discovery and testing of regulatory networks explaining morphological outcomes directly from the resultant phenotypes, producing network models as testable hypotheses explaining regeneration data. Our system integrates a formalization of the published results in planarian regeneration, an *in silico* simulator in which the patterning properties of regulatory networks can be quantitatively tested in a regeneration assay, and a machine learning module that evolves networks whose behavior in this assay optimally matches the database of planarian results. We applied our method to explain the key experiments in planarian regeneration, and discovered the first comprehensive model of anterior-posterior patterning in planaria under surgical, pharmacological, and genetic manipulations. Beyond the planarian data, our approach is readily generalizable to facilitate the discovery of testable regulatory networks in developmental biology and biomedicine, and represents the first developmental model discovered *de novo* from morphological outcomes by an automated system.

## Introduction

Advances in developmental biology and regenerative medicine require a mechanistic understanding of the generation and repair processes that construct and repair complex anatomical structures [1]. For example, a salamander can regenerate complete limbs, eyes, tails, and jaws [2]; a tail grafted to its flank will, within a few months, becomes re-patterned into a structure more appropriate to its new location—a limb [3, 4]. During metamorphosis, tadpole faces with very abnormal organ positions become transformed into normal frog faces, as each organ undergoes evolutionarily-novel movements to ensure that it ends up in the right position relative to the others [5]. Planarian flatworms regenerate their complex body from almost any surgical amputation, and cease new growth and remodeling when their correct body pattern has been restored [6]. Learning to understand and harness these high-order pattern control programs is of high importance not only to basic developmental and evolutionary biology, but also underlies the roadmap to transformative advances in regenerative medicine, birth defects, and synthetic bioengineering.

High-resolution genetic analyses are revealing an increasing number of regulatory genes, while developmental and regenerative research is producing a rich dataset of *in vivo* experimental manipulations and their resultant morphological phenotypes [7]. Unfortunately, our ability to understand and manipulate 3-dimensional patterning outcomes has not kept pace. A fundamental gap exists between the gene products experimentally identified as *necessary* for producing a morphological phenotype, and a mechanistic regulatory network that would be *sufficient* to explain exactly how and why a complex morphology is generated in the precisely correct size, shape, and orientation [8–10]. There exist individual examples of models that incorporate geometry [11–19] and attempt to understand the dynamics of patterning [20–29], but the most prevalent arrow diagrams derived from genetic experiments largely do not specify, constrain, or explain the remarkable geometry and regenerative regulation of biological systems.

Finding the mechanisms responsible for a given set of anatomical phenotypic data remains a significant challenge due to the non-linearity of many biological processes [30]. The increasing deluge of genetic data does not generally result in constructivist models that truly explain dynamic morphogenesis of living structures because it is simply too hard for human scientists to invent a model with all of the appropriate higher-order patterning properties. Indeed each additional dataset on patterning outcomes from some perturbation makes it more difficult, not easier, to come up with a model that matches all of the results. Thus, there is a clear need for automated tools to assist in the discovery of mechanistic models that explain the ever-increasing set of functional phenotypic results in the scientific literature on developmental and regenerative biology [1].

Tremendous progress has been made in developing bioinformatics tools for the reverse-engineering of dynamical models of regulatory networks from microarrays and quantitative PCR gene expression profiling data [31–43] as well as of metabolic networks from time-series concentration data [44–46]. However, these approaches produce models lacking spatial information and are not applicable to patterning and morphological experimental data. Indeed, inferring characterized regulatory networks from experimental resultant spatial patterns is exceedingly challenging due to the difficulties in robustly quantifying phenotypic data [47], evaluating spatial-temporal models with these data [48], and automatically characterizing known and unknown products and their underlying complex, non-linear interactions resulting in the desired patterning behavior [49, 50]. The gene circuit method [51–53] and subsequent automated approaches [54–63] have successfully reverse engineered a complex dynamical regulatory network from spatial data: the gap gene network controlling *Drosophila* blastoderm patterning. However, these methods are still limited to quantitative 1-dimensional gene expression data and are not amenable for morphological phenotypes resulting from surgical manipulations and genetic and pharmacological treatments that are common in developmental and regenerative biology.

No tools yet exist for mining the published datasets of experimental morphological data in regeneration and developmental biology. The complexity of anatomical and morphological data, the elaborate surgical, genetic, and pharmacological perturbation experiments, and the lack of methods to formalize in a mathematical language these data prevent us from reverse-engineering the key regulatory networks in development and regeneration. In consequence, the discovery of mechanistic regulatory networks has not kept pace with the increasing generation of phenotypic data from perturbation experiments. For example, despite over 100 years of focused attention, no quantitative model has been found that reproduces more than a few of the main features of the rich functional dataset on planarian regeneration [64]. We have learned much about the molecular pathways regulating stem cell decision-making [65, 66], but the understanding of axial polarity, morphogenesis, and persistent changes to the bodyplan [67] still lacks constructivist models. In order to make use of the ever-increasing data on patterning outcomes of genetic, pharmacological, and surgical experiments, bioinformatics must be extended to anatomy and pattern formation.

**Fig 1:**
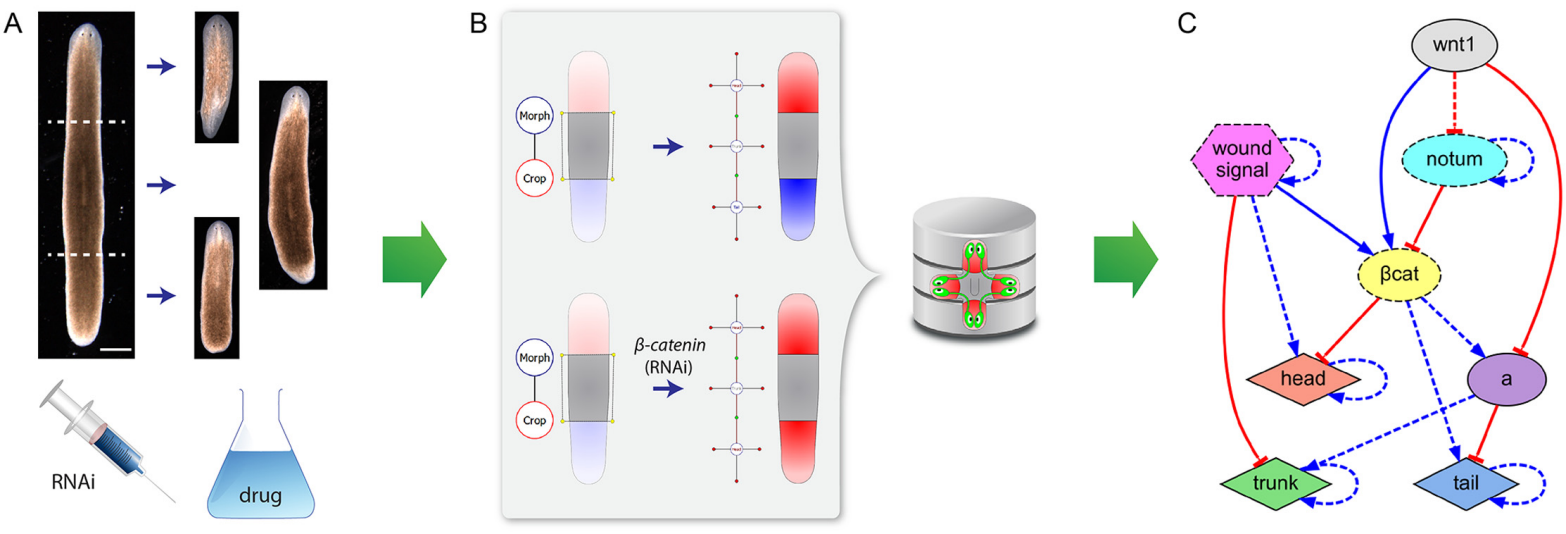
Discovering regulatory networks that explain experimental morphological data. (*A*) Surgical, genetic, and pharmacological experiments result in a set of patterning phenotypes. (*B*) Using a mathematical functional ontology, the experimental manipulations and the resultant phenotypes are formalized in a database. (*C*) Without any further knowledge beyond that dataset, the algorithm automatically infers a regulatory network that, when quantitatively modeled in a simulator, explains all the resultant phenotypes in the experiments. Discovered regulatory networks comprise specific genetic products (*β-catenin*, *notum*, *wnt1*), phenotypic products (head, trunk, tail, wound signal), and yet-unidentified products (labeled with single letters, e.g. ‘a’). Products can travel intercellularly (dashed border) or be intracellularly confined (solid border). Regulatory interactions can activate (blue lines) or repress (red lines) a product and these can be combined in a necessary (dashed lines) or sufficient (solid lines) fashion.

We present here an automated method for the discovery of regulatory networks explaining the morphological patterning results from surgical, genetic, and pharmacological perturbation experiments (Fig 1). Our system integrates a formalization of the published results in planarian regeneration, a versatile *in silico* simulator in which the patterning properties of any regulatory network can be quantitatively tested in a regeneration assay, and a machine learning module that evolves networks whose patterning behavior optimally matches the dataset of planarian results. We demonstrate that regulatory networks comprising specific biological products can be automatically inferred from phenotypic morphological data resulting from functional experiments by an evolutionary computation process. The formalized experimental descriptions of surgical manipulations, genetic and pharmacologic treatments, and resultant phenotypes are used to infer the necessary and sufficient molecular products, their interactions, and the spatial and temporal dynamics of a regulatory network explaining the given set of phenotypic experiments.

In inferring regulatory networks from phenotypic experimental data, unambiguous mathematical formalisms must be used to describe the relevant characteristics of the experimental dataset to explain (Fig 1). To this purpose, we used a functional mathematical ontology with an adequate level of abstraction for the formalization of developmental and regenerative experiments [47]. In contrast to ontologies based on natural language, our functional ontology uses mathematical language for unambiguously describing the experimental procedures as a hierarchy of elemental actions and their morphological outcomes as a set of interconnected body regions (head, trunk, and tail). Thus, the formalized experimental procedures can be reliably performed in a simulator *in silico*, and the phenotypes of the formalized experiments are amenable to automated comparison with the predictions of models. Using an evolutionary algorithm search module, our system discovered the first quantitative, constructive model that predicts the main features of planarian regeneration.

## Results

### Method to infer regulatory networks from morphological outcomes

**Fig 2:**
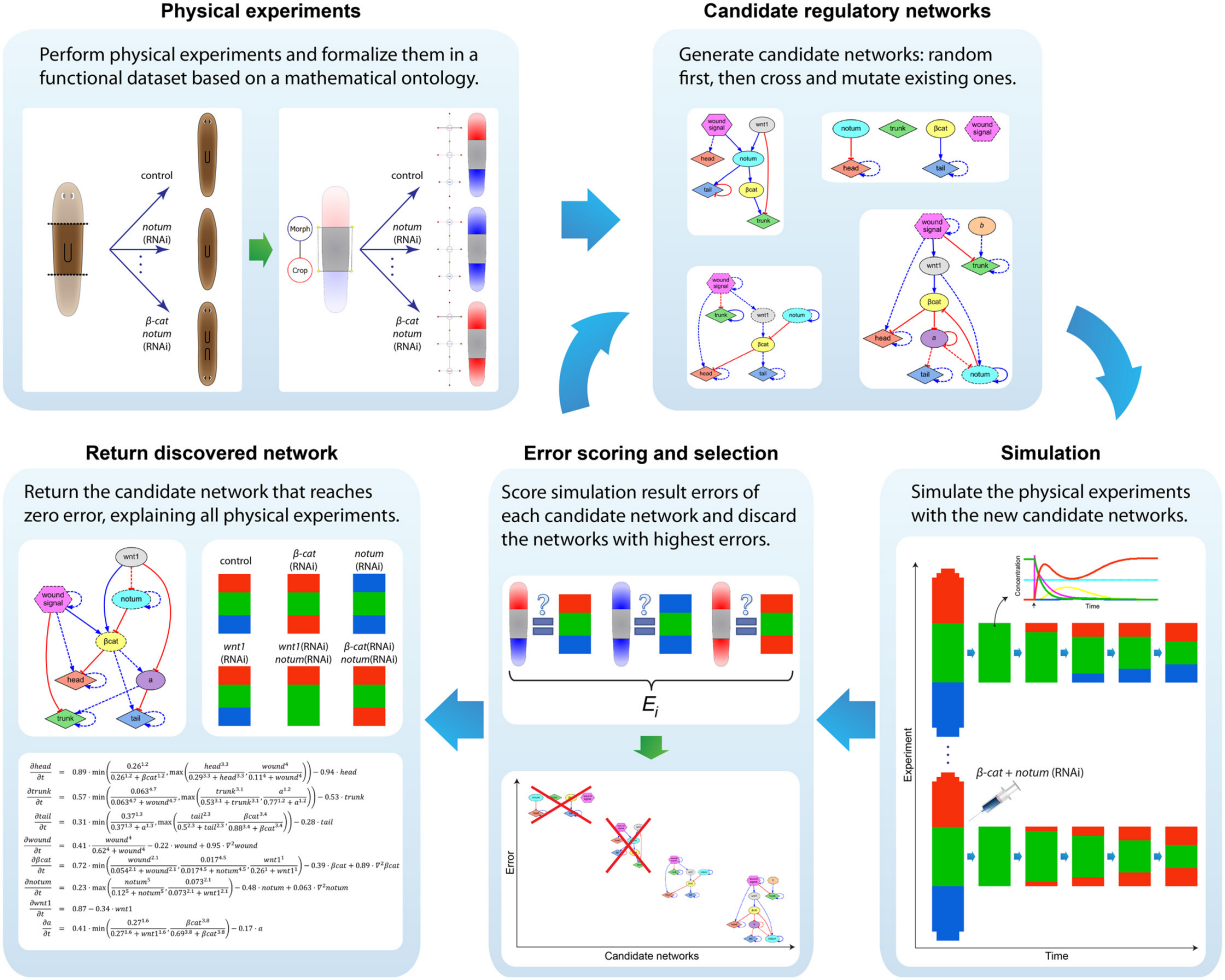
Method to infer regulatory networks from phenotype-based experiments. Taking a dataset of formalized experiments as input, the algorithm cyclically generates candidate regulatory networks, simulates the experimental manipulations, and discards the networks with the highest errors (lowest predictive power); this process is repeated until a network with zero error is found.

We developed a generalized method to infer regulatory networks from a set of formalized, morphology-based experiments (Fig 2). Focusing on the planarian regeneration data [68] for the first proof-of-principle, our goal was to identify a regulatory network that could be executed on every cell in a virtual worm such that the patterning outcomes of simulated experiments would match the published data. Based on evolutionary computation principles [69], the algorithm maintains an evolving population of candidate regulatory networks for searching the space of possible networks. The algorithm searches simultaneously for the necessary products, topology, specific regulatory interactions, and parameters of the regulatory networks, which are implemented as a non-linear system of partial differential equations. Nodes in the regulatory network can represent either signaling products or special products with a phenotypic meaning specific to the dataset (head, trunk, and tail regions in the worm).

The initial population of candidate regulatory networks is made of simple networks with random regulations and parameters. New regulatory networks are created in each generation, by combining two existent (parent) regulatory networks from the current population and probabilistically adding and removing products and regulations, and altering their parameters (see methods section). The population is cyclically updated replacing old regulatory networks with the new regulatory networks that better fit the experimental dataset. The algorithm stops when a regulatory network is found that perfectly reproduces the same resultant phenotypes in all the experiments as formalized in the input dataset.

Our system uses *in silico* experiments equivalent to the *in vivo* experiments formalized in the dataset to evaluate the predictive ability of candidate regulatory networks. For this, we implemented a simulator capable of performing the same kind of experiments formalized in the dataset, including surgical manipulations and genetic and pharmacological perturbations. An experiment stored in the dataset is simulated using a specific regulatory network in two stages: the wild-type morphology stage where the regulatory network can reach a stable state and the experimental stage where the resultant phenotypes are obtained. During the first stage, the product concentrations are initialized and the system of partial differential equations with this initial condition is numerically solved for a constant time interval. Phenotypic products are initialized to match the morphological regions pattern (head-trunk-tail) of the formalized wild-type morphology, while the signaling products are set to a continuous parameter value automatically found by the inferring method for each product. The second stage proceeds by applying the surgical manipulations and pharmacological treatments. Surgical manipulations change the system boundaries, while genetic and pharmacological treatments alter specific parameters of the differential equations corresponding to the perturbed products. Next, the new system of partial differential equations with the new initial condition and boundary is numerically solved for an additional constant time interval. The final state represents the resultant phenotype corresponding to the simulated experiment.

Thus, each candidate network model is tested in a virtual worm, under simulated experiments, to determine its patterning properties in each case. Then, to determine the quality of a candidate regulatory network, the algorithm compares the resultant phenotypes from the simulation of each experiment with real published data in our planarian database [47, 70]. To quantitatively ascertain the predictive quality of each model (how well it matches the available data), we calculate a composite error score representing how well each experiment’s final pattern matches the known result of such an experiment in real planaria. For this purpose, we implemented a phenotypic distance metric that measures how different any two morphological phenotypes are [71]. The metric calculates the average differences between the phenotypic product concentrations of the two phenotypes. The predictive error of a regulatory network is then calculated as the average phenotypic distance between the resultant phenotypes from the simulated experiments and those corresponding to the formalized experimental dataset.

### Inferred regulatory networks from experimental data

**Fig 3:**
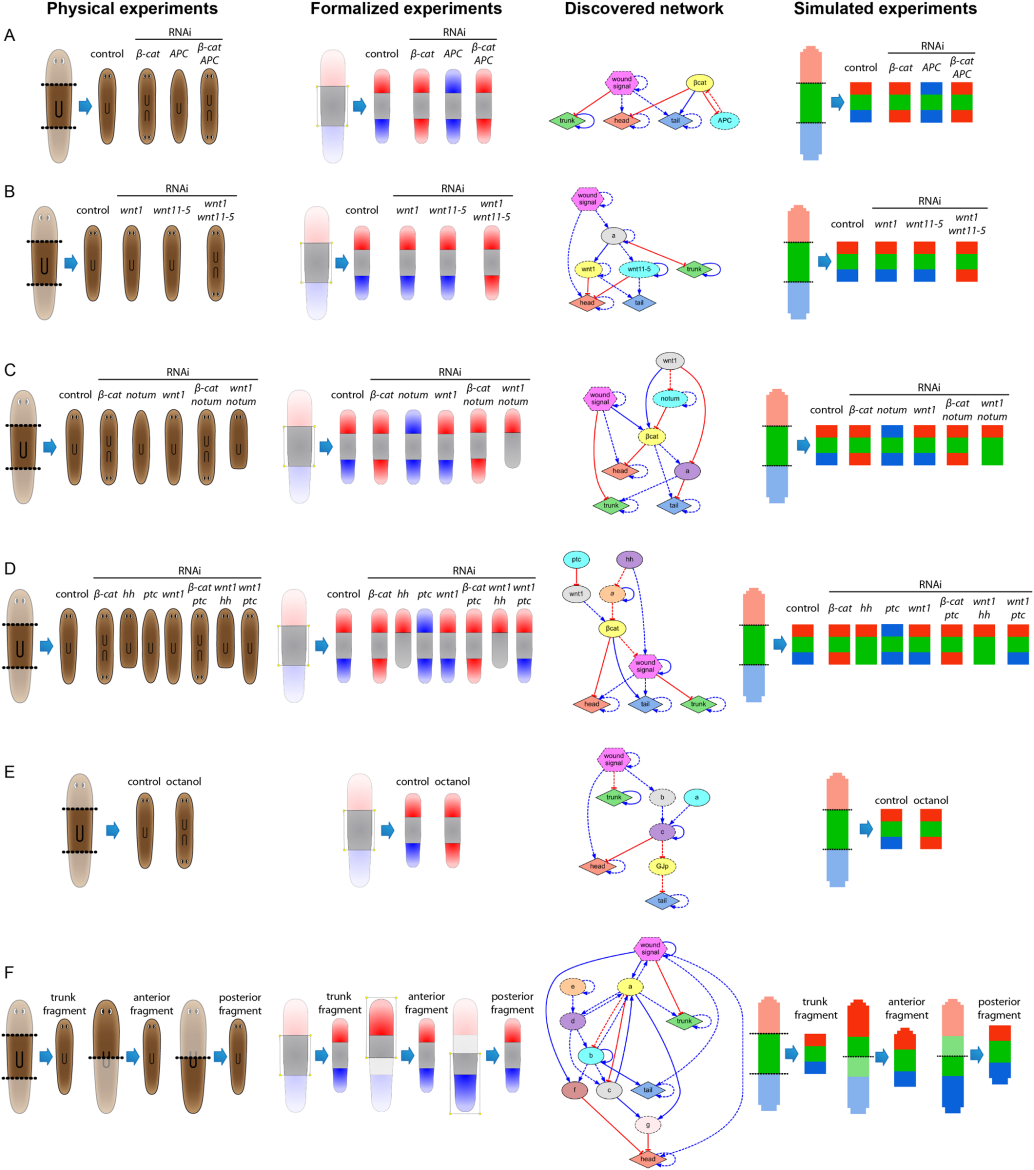
Regulatory networks inferred from experimental data formalized from the key papers of head-versus-tail planarian regeneration. The algorithm infers both the parameters and topology of the regulatory networks containing specific experimentally-perturbed products when available and unknown products when necessary, and explaining the regeneration dynamics of the correct position, shape, and proportions of the head, trunk, and tail regions of the worm for all the experiments in each dataset. (*A*) *β-catenin*/*APC* knock-down experiments. (*B*) *wnt1*/*wnt11-5* knock-down experiments [75]. (*C) β-catenin*/*notum*/*wnt1* knock-down experiments [76]; (*D*) *β-catenin*/*hh*/*wnt1*/*ptc* knock-down experiments [77]; (*E*) Gap junction communication blockage with octanol [78]; (*F*) Classical trunk/anterior/posterior fragment cuts [79].

Using this algorithmic approach, we inferred novel regulatory networks (Fig 3 and S1–S6 Movies) explaining the experimental data presented in a selection of key papers [72–79] studying the head-versus-tail regeneration decision making in the planarian flatworms *S*. *mediterranea* and *D*. *japonica*. First, we formalized datasets containing the surgical manipulations, pharmacological and genetic treatments, and their resultant experimental phenotypes for each of the selected papers. We next applied the method individually to each dataset to infer the subjacent regulatory networks explaining the experimental data presented on each of the papers. For each dataset, the algorithm found a complete system of differential equations (S1 File) that represent a regulatory network explaining the dynamical regeneration of the correct position, 2D shape, and proportions of the head, trunk, and tail regions of all the experimental phenotypes in each dataset.

Remarkably, without any prior knowledge of genetic expression patterns or regulatory interactions among genes, but using only the pharmacological, genetic, and surgical experimental perturbations and the position, shape, and proportions of their morphological outcomes (encoded as head, trunk, and tail regions), the algorithm discovered the correct known regulatory pathways of several signaling mechanisms (Fig 3). For example, the algorithm discovered the *Wnt/β-catenin* canonical regulation (Fig 3C and 3D), the inhibition of head structures and promotion of tail structures by *β-catenin* (Fig 3A, 3C and 3D), the inhibition of *β-catenin* by both *APC* (Fig 3A) and *notum* (Fig 3C), and the cryptic lack of posterior tissue re-specification (remaining as trunk) due to the knock-down of *wnt1* and *notum* (Fig 3C), *hh* (Fig 3D), or *wnt1* and *hh* (Fig 3D). In addition, several novel regulatory interactions and unidentified products were detected as necessary for the correct prediction of the experiments in the datasets.

**Fig 4:**
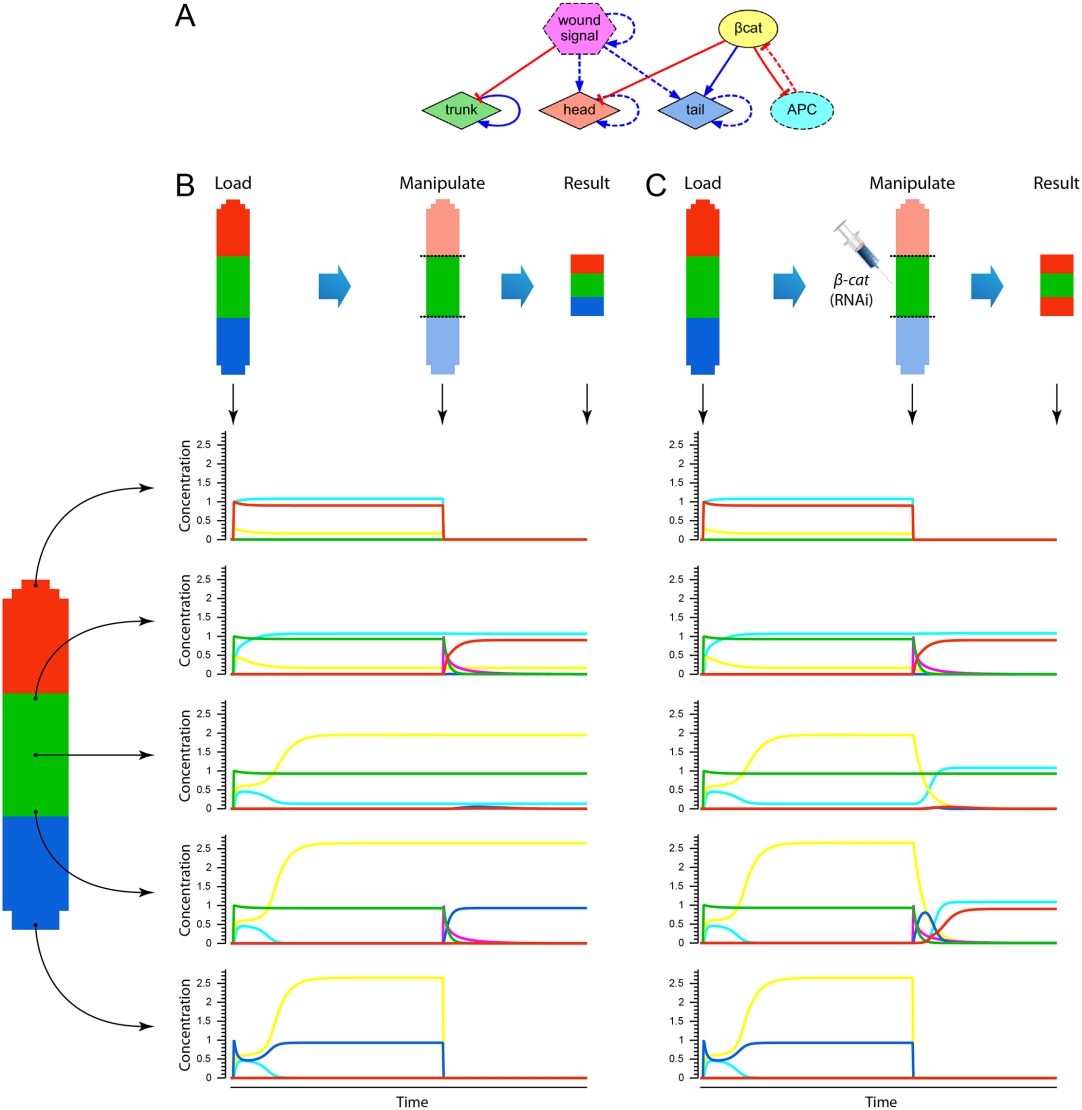
Simulation of phenotypic experiments *in silico*. (*A*) Regulatory network used in the experiment, corresponding to the inferred network shown in Fig 3A. (*B*) An experiment is performed by loading and initializing the product concentrations according to the wild-type morphology, integrating the network equations, performing the manipulation specified in the experiment, and integrating for a second time the network equations. The product concentrations over time from a selection of locations in the worm are shown. Graph colors correspond to product colors in panel A. (*C*) Knock-down of *β-catenin* (RNAi) is simulated by setting its production constant to zero, which alters the dynamics of the network and results in the regeneration of a double-head morphology.

Fig 4 shows two experiments performed *in silico* using the regulatory network discovered from the search of the model in Fig 3A. The concentration dynamics during both experiments are shown for a selection of locations in the virtual worm. In the control experiment (Fig 4B), no genetic or pharmacological perturbation was applied to the worm, resulting in the regeneration of the correct head-trunk-tail pattern. However, when *β-catenin* is blocked in the second experiment (Fig 4C), the same regulatory network predicts the regeneration of a double-head worm, which is the exact phenotype resulting from the experiments *in vivo*. The discovered regulatory network also predicted the known role of *APC* inhibiting *β-catenin*, which explains the resultant double-tail phenotype after *APC*(RNAi) (Fig 3A).

**Fig 5:**
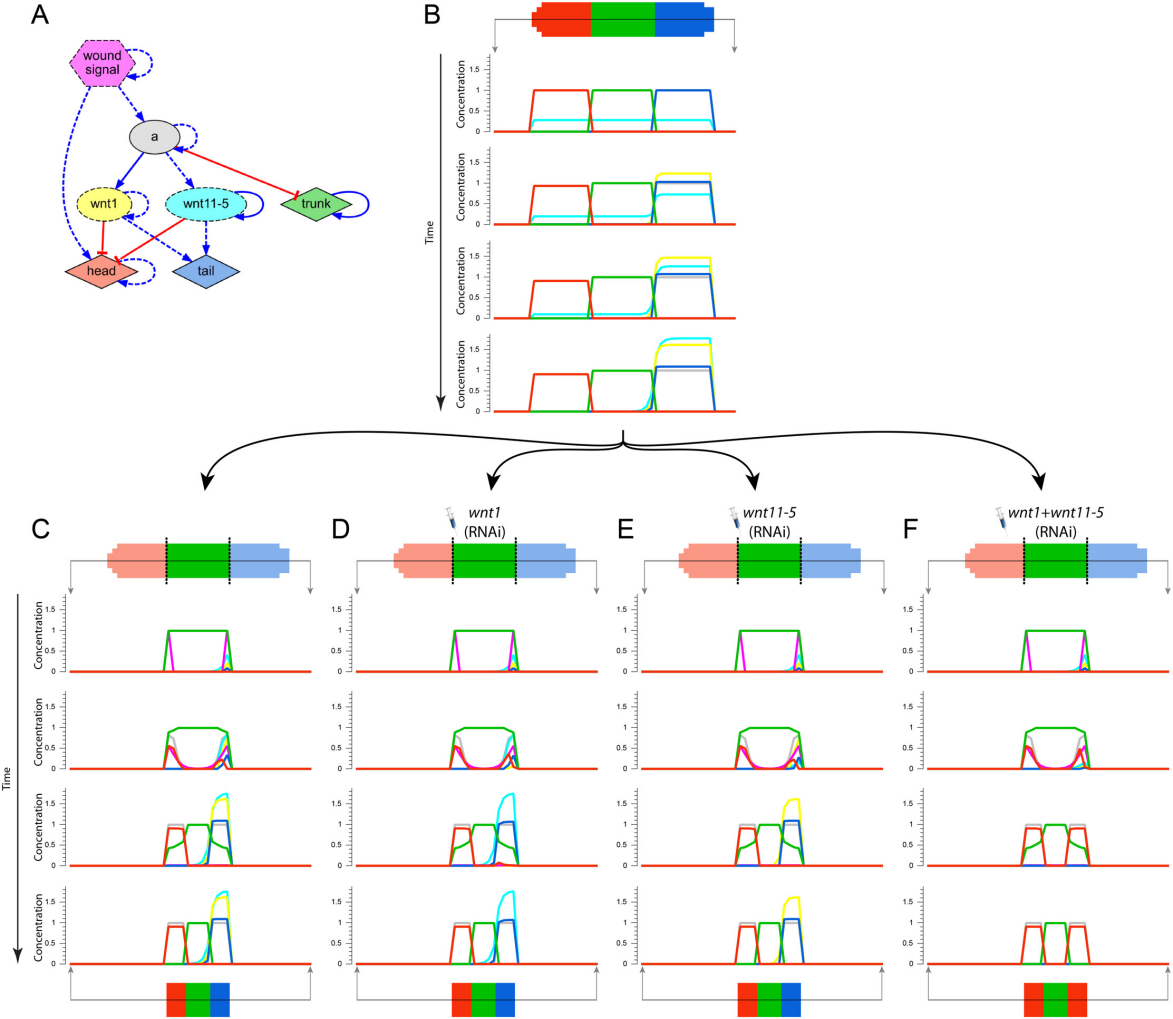
Simulation of multiple knock-down experiments. (*A*) Regulatory network used in the experiments, corresponding to the inferred network shown in Fig 3B. (*B*) First stage of the simulation, where the wild-type morphology is loaded, and the regulatory network is numerically simulated for a constant amount of time, converging to a stable state. The product concentrations of the anterior-posterior middle line are shown during different time steps. (*C-F*) Second stage of the simulation of a set of knock-down experiments. Starting with the resultant stable state in the first stage, the manipulations are performed, and the regulatory network is numerically simulated for a constant amount of time, converging to a stable state. The simulation shows how a simple cut or a single knock-down of either *wnt1* or *wnt11-5* results in the wild-type morphology, but a double knock-down of both *wnt1* and *wnt11-5* results in a double-head morphology.

Multiple knock-downs in the *wnt1/wnt11-5* regulatory pathway are necessary to perturb the resultant phenotype from a trunk fragment [75] (*wnt1* and *wnt11-5* were known as *wntP-1* and *wntP-2*, respectively [80]). When we applied the automated method to this dataset, the resultant model found consisted in a redundant modular network (Fig 3B). Fig 5 illustrates the experiments in this dataset performed *in silico* with the network automatically discovered. The regulatory network presents both *wnt1* and *wnt11-5* activating the regeneration of tail and inhibiting the regeneration of head, and both of them activated by an unknown common product. Due to this redundancy in the network design, the knock-down of either *wnt1* or *wnt11-5* results in the same phenotype than the control: the correct head-trunk-tail pattern. However, when both *wnt1* and *wnt11-5* are simultaneously knocked down, the regenerated phenotype is then a double-head worm, similarly to the phenotypes obtained *in vivo*.

**Fig 6:**
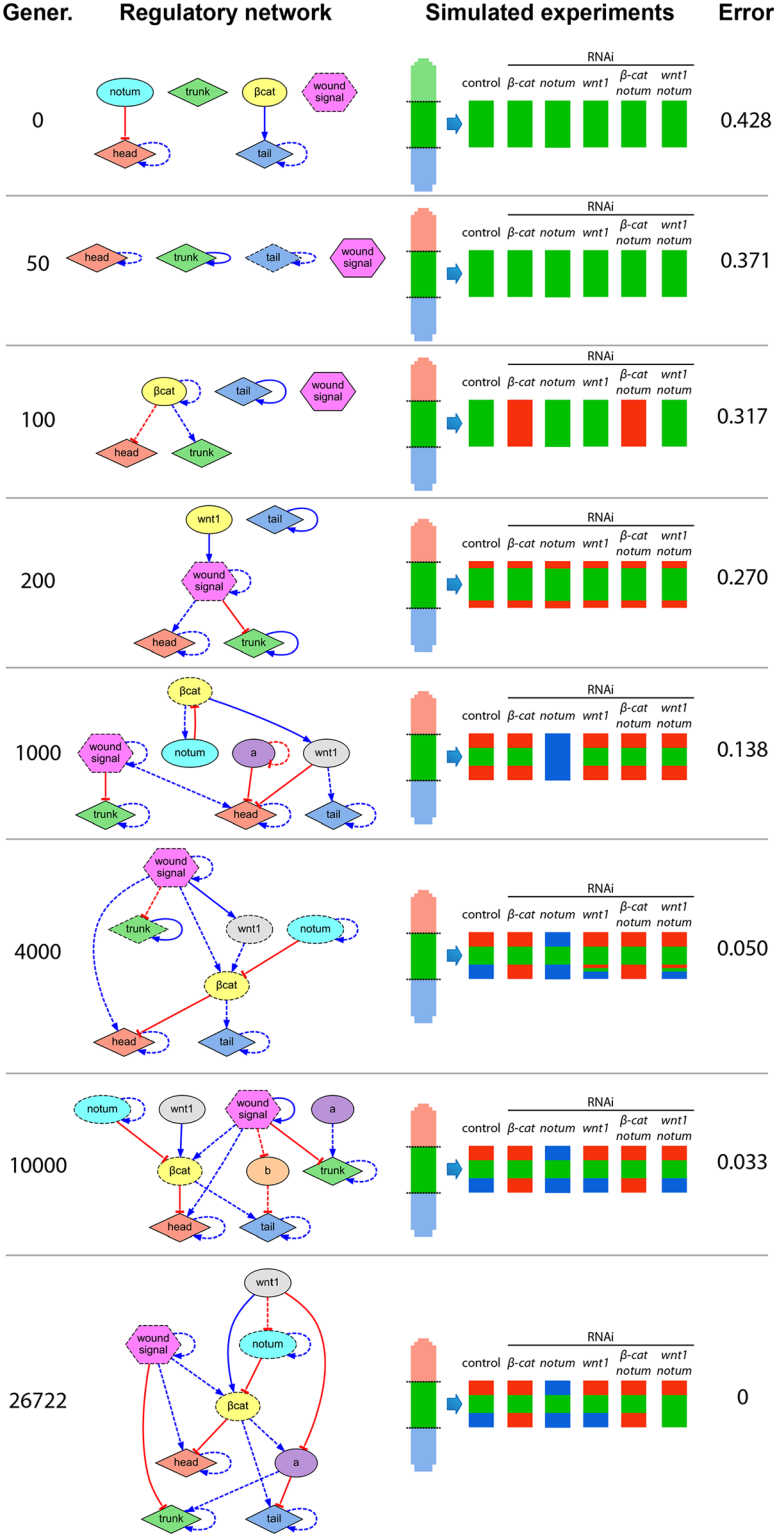
Selection of candidate networks evaluated during a search. Networks early in the search have low complexity and a limited capacity to explain the experiments in the dataset. The algorithm produces new networks by adding, deleting, and modifying products and regulations, until a network perfectly explaining all the experiments is found. The networks shown are a selection from the search of the model presented in Fig 3C.

The inferring method iteratively produces regulatory networks that better predict the experiments in the dataset. Fig 6 shows a selection of candidate regulatory networks generated during the search of the model in Fig 3C (see S1 File for the system of equations for each regulatory network). The initial random regulatory networks (generation 0) usually cannot reproduce any of the resultant phenotypes in the dataset, neither maintain the wild type morphology pattern. New candidate regulatory networks are generated by randomly combining previous networks and performing random changes, additions, and deletions, including nodes representing knocked-down genes in the experiments or unknown nodes found *de novo*. Incrementally, the new candidate networks can explain a higher number of experiments, and the final regulatory network can correctly explain all the experiments in the dataset.

**Fig 7:**
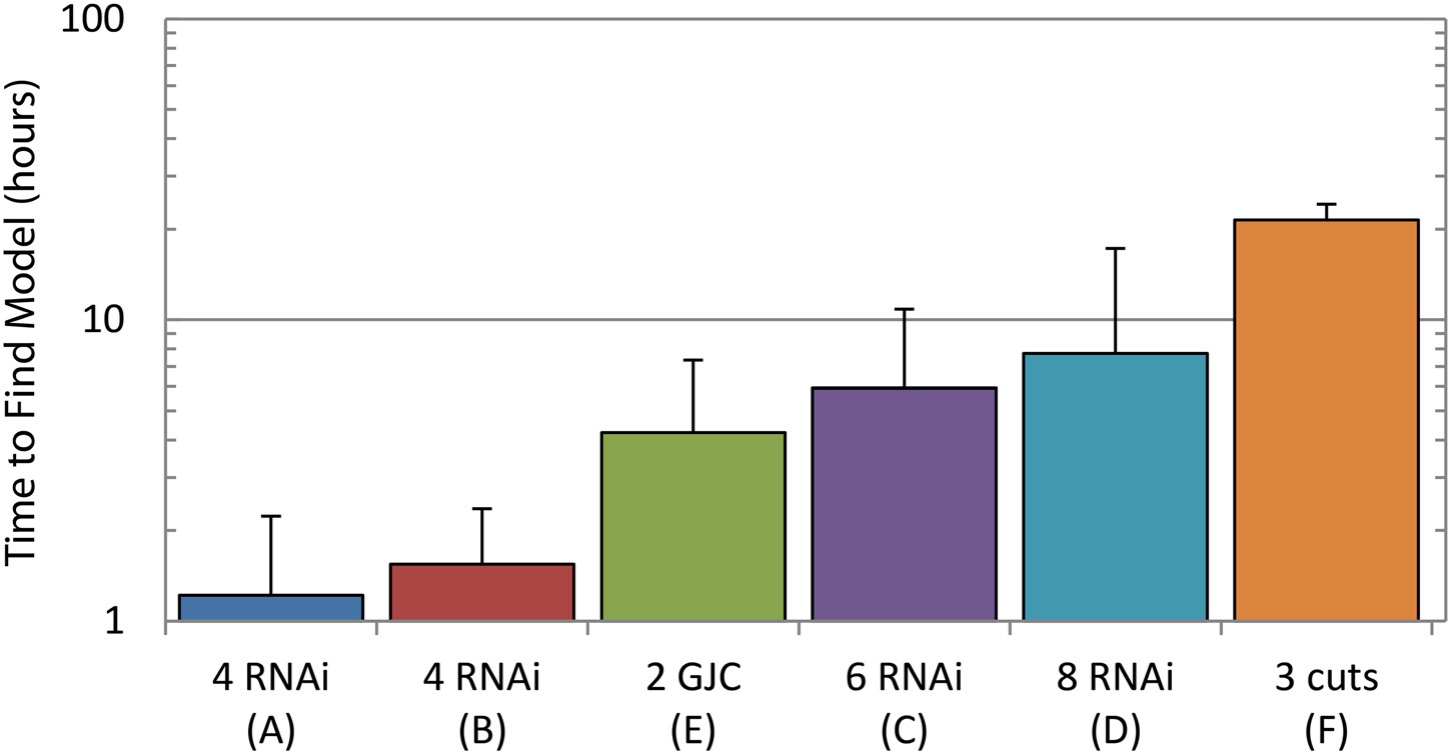
Performance of the algorithm. The computation time increases with the number of experiments in the dataset and their complexity. Knock-down experiments (RNAi) are faster to process than diffusion blocking experiments (GJC), with surgical cut experiments being the most difficult to infer. Letters refer to panels in Fig 3. Error bars denote the standard deviation.

The time to converge to a satisfactory regulatory network depends on the complexity and quantity of the experiments included in the input dataset (Fig 7). The inferring algorithm is intrinsically parallel, since the simulation and evaluation of a population of candidate regulatory networks can be done independently. Using a parallel implementation of the algorithm in a computer cluster, the time to find a regulatory network from knock-down experiments ranged from an average of one hour for four-experiment datasets to seven hours for eight-experiment datasets. Datasets with experiments blocking the diffusion of a product averaged four hours. The dataset with three classical cut experiments averaged a time to find of 21 hours, suggesting a higher difficulty in inferring *de novo* all the unknown components in the regulatory network.

We can visualize the evolution of regulatory networks during a search process by tracking the error of the best network (lowest error) in the population and its complexity (the sum of the number of products and number of regulatory interactions in the network) over time (S1 Fig). The graphs show how the initial population contains networks with low complexity and high error. Gradually over time, the error of the networks improves, while their complexity increases, until a network with zero error is found by the algorithm. During the search process, regulatory networks can evolve products and regulations that do not participate directly or indirectly in the regulation of phenotypic products, and hence do not affect the dynamics of the phenotypic products (S2 Fig). These auxiliary products are not included during the simulation of a regulatory network, but they can evolve independently through neutral mutations, and be reused at later generations by the search algorithm.

### Inferred comprehensive model of planarian regeneration

**Fig 8:**
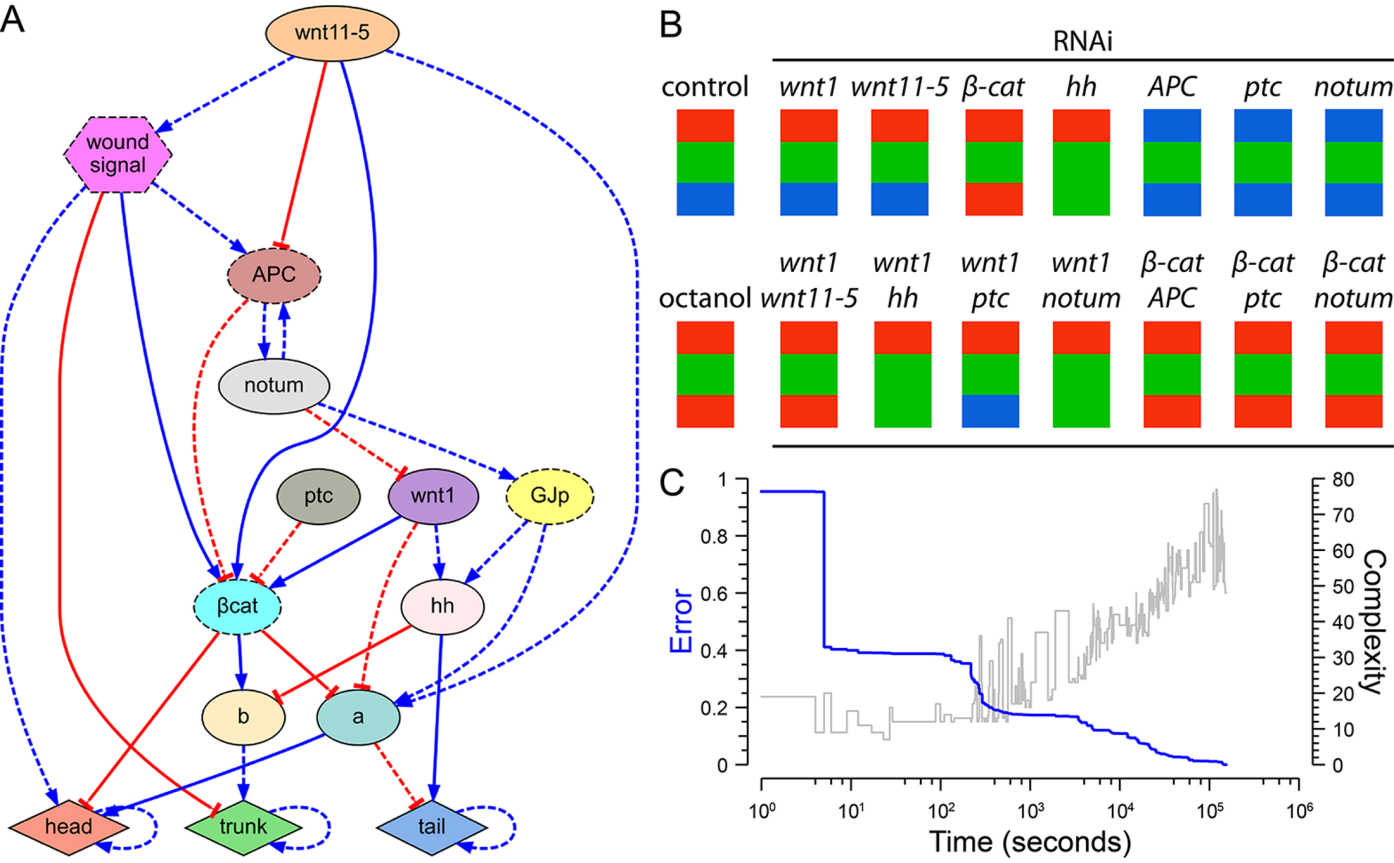
Inferred comprehensive model of planarian head-versus-tail regeneration. (*A*) Regulatory network found by the automated system, which explains the combined phenotypic experimental data of the key publications of head-trunk-tail planarian regeneration. (*B*) Simulation of the 16 experiments comprising the dataset, resulting in the same reported experimental phenotypes. (*C*) Evolution of the error and complexity (number of products and regulations) of the best regulatory network in the population over time during the algorithm search.

We next applied the algorithm to a combined experimental dataset comprising all the selected head-versus-tail planarian regeneration papers to determine whether our approach could identify a comprehensive regulatory network of planarian regeneration (Fig 8 and S7 Movie). Remarkably, after 42 hours, the algorithm returned the discovered system of equations (S1 File) representing a regulatory network that correctly predicts all 16 experiments included in the dataset. The network comprises seven known regulatory molecules inferred from knock-down experiments, one unknown gap junction-permeable diffusible product inferred from a gap junction blockage experiment, and two unknown general regulatory products. This automatically inferred regulatory network represents the most comprehensive model of planarian regeneration found to date, the only known model that mechanistically explains anterior-posterior polarity determination in planaria under many different functional experiments, and the first patterning model discovered from morphological outcomes by an automated method—a new successful application towards the augmenting of scientific discovery with artificial intelligence [81–83].

In order to characterize the two unknown regulatory products identified by the algorithm, we searched for products with similar interactions in public molecular interaction databases. Using the MiMI database [84, 85], we extracted all the known products (in *Homo sapiens*) interacting with the products that were predicted to regulate node ‘b’ (*β-catenin* and *hh*; see Fig 8) and found *hnf4* as the only common product interacting with both of them. This is thus an excellent candidate for node ‘b’, and a homolog for this gene has already been found in planaria [86]. For node ‘a’, we used the STRING database [87], and identified the Frizzled family of receptors as commonly interacting (with the highest confidence score of 0.9) with *β-catenin*, *wnt1*, and *wnt11*. Indeed, several Frizzled protein homologs have been already identified in planaria [72]; since phenotypes for each individual Frizzled gene product have not yet been uncovered by loss-of-function analyses in the literature (suggesting redundancy), our network’s node ‘a’ likely represents the regulatory actions of several of these family members as a group.

**Fig 9:**
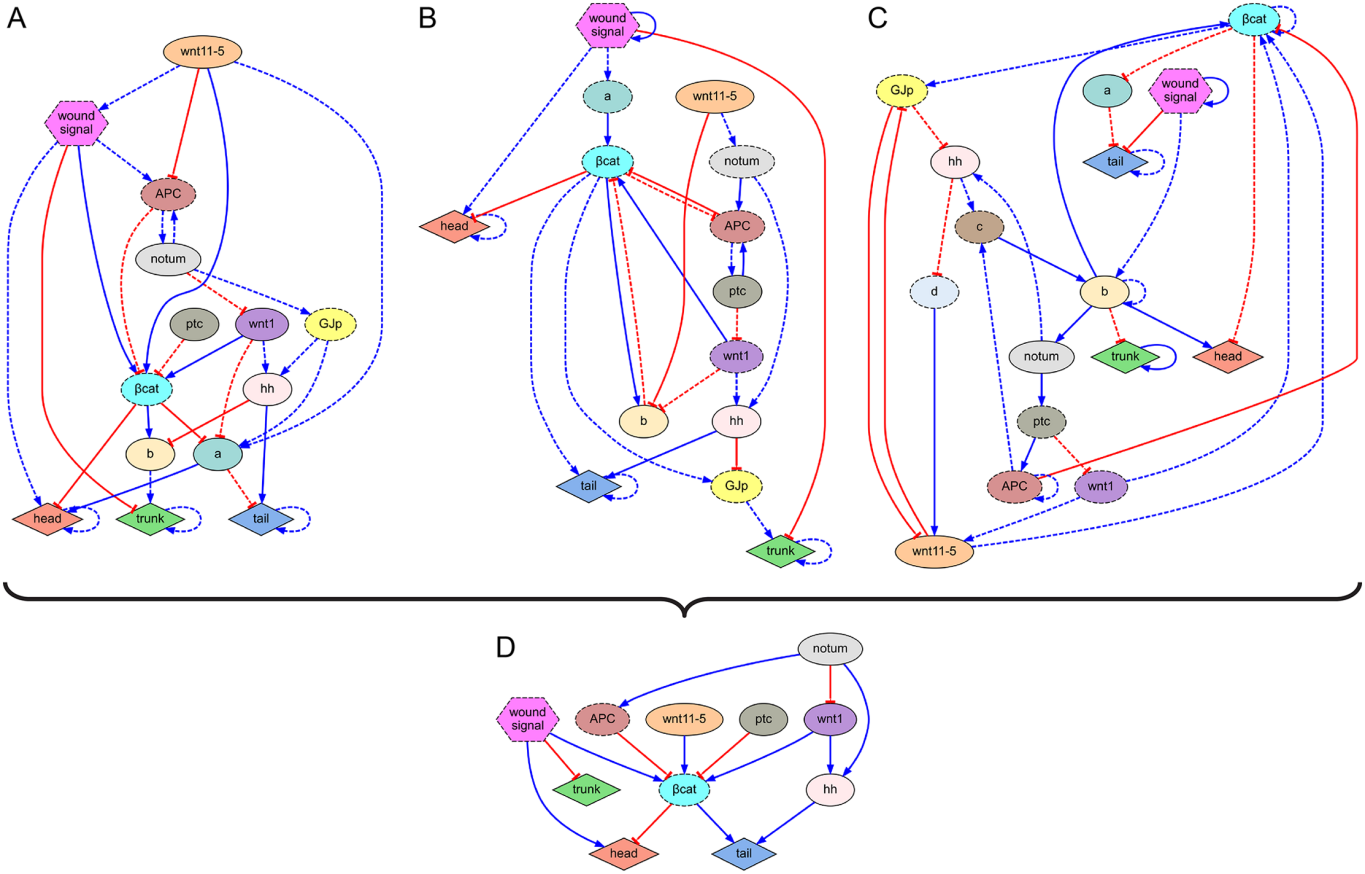
Multiple searches result in different regulatory networks with shared common pathways. (*A-C*) Regulatory networks found by the automated system during three different searches with the same 16 experiments of head-trunk-tail planarian regeneration as the input dataset. The three found networks can correctly explain all the experiments in the comprehensive dataset. (*D*) Common regulatory interactions present in the three found networks. These regulations consistently found by the automated method represent the most robust interactions that can be extracted from the comprehensive dataset.

We next tested whether some regulatory pathways were robustly found by the search method—consistently discovered by independent evolutionary searches. To this end, we performed multiple runs of the method with the same comprehensive set of experiments. These searches resulted in three different regulatory networks that can correctly reproduce the complete set of experiments (Fig 9A–9C; A being the most parsimonious network that was presented in Fig 8). All the regulations shared by these three networks are seen together in a common subnetwork (Fig 9D). Remarkably, 14 regulatory interactions were consistently found by the search method, suggesting that these relationships are the most important interactions explaining the comprehensive dataset of experiments.

**Fig 10:**
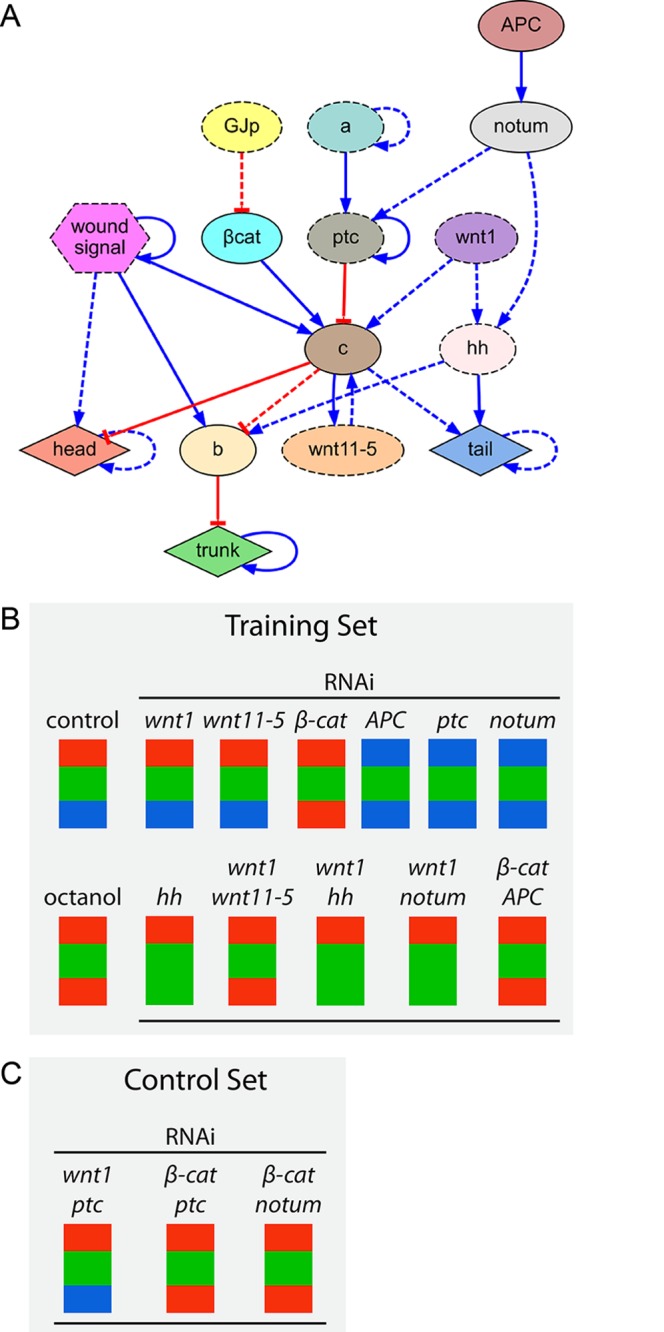
A subtraction control experiment with the automated method results in a regulatory network that can correctly predict three novel experiments not included during the search. (*A*) Regulatory network found by the automated system from a reduced comprehensive dataset excluding three experiments. (*B*) Simulations of the reduced dataset of experiments used during the search with the found network result in the regeneration of the correct phenotypes. (*C*) Simulations of the three novel experiments not included during the search with the found network result in the exact correct phenotypes obtained *in vivo*.

Finally, we tested the robustness and predictive ability of the regulatory networks found by our automated method in an experiment designed to test the predictive value of the discovered models for data they had never seen (not included in the search process). We omitted three key experiments from the comprehensive dataset, and used the automated method to find a regulatory network that could correctly reproduce this reduced (partial) dataset (Fig 10A and 10B). Crucially, the found regulatory network correctly predicted the outcomes of these three novel experiments—the model correctly explained the outcomes that were not known to it during the search (Fig 10C). These results validate the ability of the automated search method to find regulatory networks capable of not only explaining the resultant phenotypes from the experiments performed *in vivo* included in the learning dataset, but also of predicting the resultant phenotypes from novel experiments. We conclude that the networks uncovered by this system have predictive value for novel results, in addition to helping to understand existing data from which they were extracted.

## Discussion

Our system addresses the gap between the wet-lab discovery of genetic regulatory interactions and an understanding of the dynamic patterning behavior of regenerative systems. Comprising (1) a formalized database of functional patterning outcomes in the planarian literature, (2) a simulator in which any (human- or computer-derived) regulatory model can be evaluated for fit to known anatomical data, and (3) a network discovery machine learning method, this system is a first step towards a new bioinformatics of shape. These three modules are integrated into a workflow designed to help human scientists discover mechanistic, constructivist models that optimally match the ever-growing dataset of regeneration data.

Our results demonstrate the discovery of regulatory networks directly from formalized experimental morphological data with the use of an automated computational algorithm—the first automated linkage of morphological output and molecular-genetic underpinnings. With no prior information beyond the input dataset of functional outcomes of surgical, genetic, and pharmacological experiments, the method is capable of identifying the necessary biochemical products and their regulations and parameters that form a system of partial differential equations explaining the resultant phenotypes from the dataset. The networks discovered by our system represent immediately testable hypotheses for the control algorithms underlying regeneration.

Our method improves the current state of the art for reverse-engineering dynamic regulatory networks in several areas. Foremost, our method is the first to be applicable to data containing *morphological* outcomes and surgical perturbations, which is essential for the regeneration field. Current methods are limited to inferring networks from dimensionless gene expression profiling data or 1-dimensional expression data resulting from genetic perturbations. In contrast, our method is flexible enough to extract regulatory networks directly from resultant 2-dimensional morphological patterning outcomes and to process a wide array of experimental perturbations, including surgical manipulations, pharmacological treatments, and genetic knock-downs. To this end, we implemented a whole-body developmental simulator that differs from current approaches [88–91] in that the input is a formalization of both the experimental surgical and genetic perturbations to perform and the dynamical regulatory model to test. This allows our method to be applicable to the reverse engineering of regulatory networks from the morphological outcomes of developmental systems, previously out of the range of automated inference methods, including the large experimental dataset of regenerative model organisms lacking mechanistic dynamical explanations.

Importantly, our method can infer regulatory networks containing not only the specific products and genes perturbed in the input experimental dataset, but also discover completely *de novo* unknown products detected as necessary to explain the resultant phenotypes: predict their existence, functional roles in the network, and properties of interaction with known molecular components. This makes our approach applicable to even datasets with perturbations affecting unknown mechanisms, as well as datasets lacking all the experimental perturbations necessary to explain all the experimental data. In this way, our method can infer regulatory pathways not apparent from the input dataset and novel interactions not reported in the literature, whose yet-to-be-characterized products can be identified from the multiple interactome databases available in the literature—these inferences then serve as predictions of the model which can be empirically tested. We are currently implementing an automated method to characterize such unknown products.

The inferred regulatory networks by our method contain more versatile regulatory interactions than previous approaches. Due to their capability to model a diverse set of biological regulations, we employed Hill functions to model the regulation between two products. Using two parameters per interaction (the Hill coefficient and the disassociation constant), the model can accommodate a richer variety of non-linear interactions compared to linear and one-parameter non-linear functions. Furthermore, the regulatory networks inferred by our method improve current approaches by permitting different types of aggregated interactions between multiple regulations for a single product, such as necessary regulations (both regulators are required), sufficient regulations (any regulator is enough), or any combination of them. The high flexibility of the inferred regulatory networks makes our method a very versatile approach.

The discovered regulatory networks reveal several interesting properties of the inference method. Networks matching many functional experiments that quantitatively and qualitatively explain regeneration of anatomical polarity—which had eluded human scientists—could be discovered in acceptable time by an automatic search performed by a computer. Surprisingly, the fully parameterized regulatory networks that were identified by this process are not highly complex tangles, but are similar in complexity to qualitative models proposed by human scientists in the literature and thus readily understandable. Moreover, the discovered networks contain only a few to-be-identified gene products, which facilitate their identification from known interactome data. Indeed, we could manually identify the two gene unknown products found by our method using publicly available molecular interaction databases. Currently, we are developing an automated method to facilitate the characterization of such products. Precise predictions can readily be made from the simulation of novel experiments with the discovered networks, guiding in the design of the best next set of experimental manipulations.

The comprehensive model of planarian regeneration reverse-engineered by our method represents the first quantitative model able to recapitulate regeneration under genetic knock-downs, pharmacological treatments, and surgical manipulations. Unlike conventional arrow diagrams derived from molecular genetic experiments, this system identifies models that not only include necessary components (without which regeneration cannot occur normally), but are fully-specified as a constructive model showing which dynamics are sufficient to give rise to the remarkable pattern homeostasis of planaria. Most models of regeneration are based on generalized mechanisms and do not consider the specific dynamic regulatory mechanisms or network topology necessary to precisely recapitulate the observed patterning phenotypes [92–96]. Meinhardt’s pioneering work on the mechanisms of pattern formation represents the only dynamic models of planarian regeneration proposed to date, based on reaction-diffusion mechanisms and able to recapitulate the head-versus-tail polarity regeneration and midline formation [23, 64, 97, 98]. However, this approach was purely numerical as a proof of the general dynamic mathematical principles, without characterizing any of the regulatory products, and hence accounting only for surgical amputations. Our model was inferred directly from experimental data and includes particular genetic regulatory components able to precisely predict genetic and pharmacological interventions in addition to surgical manipulations. Hence, the models inferred with our method can be used to predict the morphological outcomes in specific genetic knock-downs.

The method can identify those interactions most strongly implied by the dataset, by performing multiple searches and extracting the common pathways found in the resultant set of regulatory networks. Interestingly, the consensus model found in this way includes most of the genetic regulations of head vs. tail planarian regeneration published in the field to date, as well as novel genetic regulations only discovered recently in other model organisms, such as the inhibition of *wnt* by *notum* [99]. Furthermore, the method can be used as a generable protocol for automatically finding the less-universal regulatory interactions inferred from the data, and for automatically suggesting additional perturbations for *in vivo* experimental testing. Importantly, the robustness of the method to infer predictive regulatory networks was validated with a subtraction control test, which successfully produced a regulatory network that not only predicted all the experiments in the dataset used during the search, but also predicted the exact resultant phenotypes from a set of new *in vivo* experiments that were not part of the search process. In summary, these results validate the capacity of our method to reverse engineer robust regulatory networks with a high predictive power.

Although our method has produced the most comprehensive model of planarian regeneration to date, it contains several limitations. We have restricted our experimental formalization and simulation to 2-dimensional spatial data; thus, the discovered models do not yet address the regulatory mechanisms necessary to specify the dorsoventral axis patterning in planaria [100–103], or the detailed patterning of individual internal organs. In addition, the discovered models are deterministic, and do not account for the stochasticity shown by some partial penetrance phenotypes. Adding a stochastic component to such equations does not represent any technical difficulty; however, the computational requirements of the method to quantify the frequency for each of the possible resultant phenotypes in each experiment would increase by several-fold. Because the basic paradigm is fundamentally very flexible, future work will address these limitations, leading to further improvements in the ability to reverse engineer models that are more complete, including specific modeling of the numerous cellular mechanisms that physically implement such outcomes, such as cell migration, division, differentiation, or apoptosis.

Our approach is broadly applicable to any model system whose experimental procedures and anatomical outcomes can be formalized [104, 105] and can readily be extended to other problems in morphogenesis, including embryonic development or the programmed self-assembly of hybrid systems such as bioinspired robots [106–109]. The models discovered with this method allow the identification of the key mechanisms and the major regulatory products, including those directly perturbed during the experiments as well as-yet unidentified necessary products, explaining the resultant experimental phenotypes. Such models are required for the identification of intervention strategies to produce desired changes in large-scale shape, for birth defects, regenerative medicine, or synthetic bioengineering research. Our method represents a proof of principle towards the use of evolutionary search and quantitative spatial simulation to help constructively understand complex morphological outcomes in embryogenesis, regeneration, and synthetic bioengineering.

## Methods

### Formalizing experiments and morphologies

We created the input datasets of formalized experiments for the automated search algorithm with the software tool Planform [70]. Planform uses a functional ontology based on mathematical graphs [47], a set of interconnected nodes [110], to unambiguously describe the main characteristics of the morphology, including the overall shape and the location of specific phenotypic regions (head, trunk, and tail regions in the worm). Experimental procedures are described in the ontology as a nested set of basic operations, including amputations, cuts, genetic/pharmacological perturbations, and their parameters. Using the graphical user interface, we created a separated dataset with the phenotypic experiments presented in each of the main publications of head-versus-tail planarian regeneration [72–79], and an additional dataset including all the experiments together.

### Mathematical model of developmental regulatory networks

To apply an automated discovery system capable of finding complex spatial and temporal dynamic networks, we modeled the behavior of gene, protein, and metabolite regulatory network with a system of nonlinear partial differential equations (PDE). Products can act as intercellular signals or be confined intracellularly, decay with time, and be activated or inhibited by other products in the regulatory network. Each product can be regulated by several other products, where interactions can be combined in either a necessary or sufficient fashion.

A regulatory network is made of phenotypic products and signaling products. Phenotypic products represent phenotypic regions in the organism, and as such cannot regulate other products. In this way, morphological features of the phenotype are abstracted as a single product, resulting in inferred regulatory networks centered on the signaling mechanisms and not the molecular details to form specific morphological features. For example, full-body worm phenotypic data are formalized using head, trunk, and tail regions, whereas the inferred networks employ corresponding specific products representing head, trunk, and tail outcomes.

Signaling products can regulate other products, and they can represent the product of specific genes (such as *β-catenin* or *wnt1*) inferred from the perturbation experiments in the dataset, or be found *de novo* as necessary by the search algorithm. In addition, special products are used to model specific aspects of an experiment. In particular, we implemented a wound signal product, which is produced in the area adjacent to a surgical cut during an experiment.

Each equation in the system models the production rate of a product as the linear relation between a production term, a decay term, and a diffusion term. The production term is modeled with a combination of Hill functions, a widely-used nonlinear model of biochemical interactions and genetic regulation [111]. Each Hill function models the activation or repression of a product by another product (including itself). A product can be regulated by several regulatory interactions simultaneously, and these interactions can be grouped in a necessary (both regulators are required to produce the regulated product), sufficient (one regulator is enough to produce the regulated product), or any combination of them. Sufficient interactions are grouped together in a *max* operator, while necessary interactions are grouped together in a *min* operator; the set of sufficient interactions is considered as a necessary interaction by itself, and hence it is included inside the *min* operator. The rate or production is modulated by a production constant, which multiples the result of the combined Hill functions regulation. Products decay in an exponential fashion. Thus, the decay term is modeled with a decay constant multiplying the product current concentration. Intercellular signaling mechanisms are essential in the regulation of developmental and regenerative processes. We modeled the propagation of intercellular signals as a diffusion term in the differential equation, modulated by a diffusion constant. This allows the implementation of products that can propagate intercellularly, carrying signals regulating other products. The diffusion constant of a product can be zero, in which case the product is considered exclusively intracellular.

The following equation illustrates a model of the production of product *a* as regulated by two necessary products (activator *b* and inhibitor *c*) and two sufficient products (activator *d* and inhibitor *e*):

$$\frac{\partial a}{\partial t}=\rho_{a}\text{min}\left(\frac{b^{\eta_{1}}}{\alpha_{1}{}^{\eta_{1}}+b^{\eta_{1}}},\frac{\alpha_{2}{}^{\eta_{2}}}{\alpha_{2}{}^{\eta_{2}}+c^{\eta_{2}}},\text{max}\left(\frac{d^{\eta_{3}}}{\alpha_{3}{}^{\eta_{3}}+d^{\eta_{3}}},\frac{\alpha_{4}{}^{\eta_{4}}}{\alpha_{4}{}^{\eta_{4}}+e^{\eta_{4}}}\right)\right)-\lambda_{a}a+D_{a}\nabla^{2}a$$

where *ρ*_*a*_ is the production constant, *η*_*i*_ are the Hill coefficients, *α*_*i*_ are the dissociation constants in the Hill functions, *λ*_*a*_ is the decay constant, and *D*_*a*_ is the diffusion constant.

In summary, each product of a regulatory network is defined according to four parameters (production, decay, and diffusion constants, and the initial concentration value), while each regulatory interaction is defined with three parameters (Hill coefficient, dissociation constant, and whether the regulation is necessary or sufficient). The values of all the parameters are automatically inferred by the search algorithm.

### Performing phenotypic experiments *in silico*

We implemented a simulator able to load morphological phenotypes and perform surgical, genetic, and pharmacological experiments formalized with the functional ontology. The simulator takes as input a formalized experiment with the functional ontology and a regulatory network described with a system of PDEs. The simulator outputs the resultant morphology after numerically integrating the PDE system and performing *in silico* the formalized experiment. Due to the dynamic boundaries of a developmental and regenerative simulated organism, we implemented an Euler finite difference method [112] to integrate the set of PDEs corresponding to a regulatory network.

The simulator performs an experiment in two stages. During the first stage, the original wild-type morphology is loaded into the simulator, the initial product concentrations are set according to the model, and the regulatory network defined in the PDE system is integrated for a fixed amount of time. This first stage allows the dynamical system to converge into a steady state, which will be used for the initial state in the second stage. A formalized morphology and regulatory network is loaded into the simulator by setting the initial concentration value for every product in the regulatory network. The concentration of phenotypic products (head, trunk, and tail in the worm dataset) is initialized according to the corresponding phenotypic regions. For example, the positions corresponding with a head region will be initialized with head product concentration of 1.0 and trunk and tail product concentrations of 0.0, whereas the positions corresponding with a trunk region will be initialized with trunk product concentration of 1.0 and head and tail product concentrations of 0.0. Signaling products are initially set homogenously according to either a numerical parameter for each product between 0.0 and 1.0 stored in the model or indicating a configuration similar to a phenotypic product.

During the second stage of the simulation, the surgical manipulations and genetic and pharmacologic perturbations are performed and the PDE system is integrated for another fix amount of time. The final state of the system is the resultant morphology of the *in silico* experiment. Surgical manipulations change the boundary of the system and set the concentration of all products outside of the new boundaries to zero. A genetic knock-down (RNAi) will eliminate all the activation regulations of the corresponding product. Pharmacological treatments (octanol) will set the corresponding product diffusion constant to zero, simulating a block of gap junction channels.

### Calculating the error of a regulatory network

To calculate the error (predictive power) of a regulatory network, the resultant phenotypes of the simulated experiments using the network are scored by comparing them with the resultant phenotypes from the physical experiments. For this end, we implemented a distance metric between phenotypes.

Wild type planarians can vary their size by about an order of magnitude due to feeding and starvation [113], a common situation during regenerative experiments where worms may lack the ability to feed. In consequence, we made the distance metric between phenotypes tolerant to small variations between phenotypes. More precisely, the phenotypic metric is invariant in scale, for which the phenotypes are first centered and scaled before comparison. In addition, we included a concentration tolerance parameter (*ε*) and a radius tolerance parameter (*r*) within the metric, as defined below, which even out small differences between phenotypes.

The goal of the search algorithm is to find regulatory networks that produce stable phenotypes, and not transient states that are only temporally similar to the resultant phenotypes of the physical experiments. To bias the search towards stable networks, we included a concentration change penalty that is applied when the maximum concentration change in the last time step of a simulation is higher that certain parameter threshold (*μ*).

We then define a Euclidian distance between two locations **a** and **b** that measures the squared averaged distance between a set *p* of phenotypic products within a tolerance *ε*:

$$∥a-b{∥}_{\varepsilon }=\sqrt{\underset{p\epsilon P}{\sum }{\left({\left({\left[p\right]}_{a}-{\left[p\right]}_{b}\right)}^{2}-\varepsilon \right)}^{+}}$$

where [*p*]_**a**_ and [*p*]_**b**_ are the concentrations of product *p* in the locations **a** and **b**, respectively.

Then, we define a distance metric between two phenotypes *A* and *B* of size w-h as the mean logarithmic minimum distance between every location **a** of phenotype *A* and every location **b** inside a radius *r* from **a** of phenotype *B*:

$$d\left(A,B\right)=\frac{1}{w\cdot h}\overset{w}{\underset{i=1}{\sum }}\overset{h}{\underset{j=1}{\sum }}\text{log}\left(1+\underset{\delta ,\theta \in \left(-r,r\right)}{\text{min}}∥a_{i,j}-b_{i+\delta ,j+\theta }{∥}_{\varepsilon }\right)$$

Finally, we define the error of a regulatory network model *M* for a set *E* of *n* experiments as:

$$\text{error}\left(M,E\right)=\frac{1}{n}\overset{n}{\underset{i=1}{\sum }}\left(d\left(\Psi_{e_{i}}^{M},{\text{P}}_{e_{i}}\right)+{\left(\Delta_{e_{i}}^{M}-\mu \right)}^{+}\right)$$

where $\Psi_{e_{i}}^{M}$ is the resultant phenotype of simulating experiment e_*i*_ with the model *M*, $P_{e}{}_{{}_{i}}$ is the resultant phenotype from the physical experiment e_*i*_, $\Delta_{e_{i}}^{M}$ is the average concentration change in the last time step of simulating experiment e_*i*_ with the model *M*, and *μ* is the penalty concentration change threshold. The error of a network is calculated with the set of experiments formalized in the input dataset, plus an additional experiment with no surgical manipulation or perturbation to assure that a discovered regulatory network maintains the correct wild type morphology in the absence of any perturbation.

### Searching for regulatory networks

Having an automated measurement of the error of a given regulatory network model for a set of formalized experiments, we then implemented an optimization method to search for models that minimize the error. Our method is flexible enough to find the parameters, the topology, and the necessary products of the network.

We employed an evolutionary algorithm [114] approach to search for regulatory networks, where a population of candidate networks evolve in parallel until a network with zero error is found. The initial population comprises random networks with random parameters and regulations between the phenotypic products, the wound product, and the genetic and pharmacological perturbed products from the set of experiments to search.

New regulatory networks are produced from existing ones through crossover and mutation operators. A crossover mixes randomly two networks to produce two new networks. Products that are in common between the two networks are copied to the new networks randomly, each network receiving one of each product, while products not shared are distributed randomly between the two new networks. Products are copied to a new regulatory network together with their regulatory links. If the regulatory product of a copied link does not exist in the new network, it is substituted randomly by another regulatory product.

Mutations alter the regulatory network randomly. Each product or link parameter can be substituted by a random value with 1% probability. Products and links are duplicated with 1% probability. After duplicating a product, a new regulatory link and a new regulator link is created for the new product. After duplicating a link, the regulated and regulator products are chosen randomly. Products and links can be deleted with 1.5% probability, except phenotypic and perturbed products in the experiments, which cannot be deleted. These evolutionary parameters are not optimized; however, a higher probability of deletion with respect duplication is necessary to bias the evolution towards simpler networks and prevent bloating [115].

The evolutionary algorithm stops when a network with zero error is found and the complexity (number of products and links) of the simpler network with zero error have not been decreased for a certain number of generations. This extra evolutionary time is used to simplify the best network found, since the mutation operators are biased towards simpler networks.

### Evolutionary algorithm implementation

Since new regulatory networks in a population can be simulated and evaluated independently, we implemented a parallel version of our evolutionary algorithm in a cluster computer using 256 cores. We used an island distribution approach [116], which improves performance and preserves genetic diversity by using many independently-evolving subpopulations. We used 32 parallel subpopulations with 64 regulatory networks each. For every 250 generations on average, all subpopulations are randomly paired and their regulatory networks are shuffled randomly; this compensates the trend for a single suboptimal regulatory network to saturate a single subpopulation.

We used the deterministic crowding selection method [117] with 75% crossover, 1% parameter change mutation, 1% duplication mutation, and 1.5% deletion mutation. All the parameters in the regulatory network can vary in the range (0,1), except the Hill coefficient, which can vary in the range (1,5). To calculate the regulatory network error, we used an Euclidian distance tolerance *ε* of 0.1, a distance comparison radius *r* of 2, and a penalty concentration change threshold of 10^–4^. We used 250 extra generations in the criteria to stop the algorithm after a network with zero error is found.

The simulation and search method was implemented in C++ using the Standard Library and the Eigen library (http://eigen.tuxfamily.org). Visualizations used the Qt libraries (The Qt Company Ltd.) and the Qwt library (Uwe Rathmann and Josef Wilgen). The software is freely available at *http://www.daniel-lobo.com/planarianmodels*.

## Supporting Information

S1 Fig Error and complexity over time of the best regulatory network of several search processes. (*A-F*) Each panel corresponds with the evolutionary search that resulted in the finding of the regulatory networks shown in Fig 3A–3F, respectively.
(TIF)

S2 Fig Regulatory networks can include auxiliary products and regulations that do not affect the dynamics of the network but can evolve through neutral mutations during the search. (*A-F*) Final regulatory networks including known morphological and genetic products, novel products (labeled with letters), and auxiliary products (numbered, light grey) corresponding to the inferred networks shown in Fig 3A–3F, respectively. Both the auxiliary products and novel products are found *de novo* by the search algorithm; however, in contrast to the novel products, the auxiliary products do not have regulatory interactions affecting directly or indirectly morphological outcomes (head, trunk, and tail products) and hence are not considered part of the final discovered signaling network.
(TIF)

S1 Movie Simulation of β-catenin/APC regulation model. (MP4)

S2 Movie Simulation of wnt1/wnt11-5 regulation model. (MP4)

S3 Movie Simulation of β-catenin/notum/wnt1 regulation model. (MP4)

S4 Movie Simulation of β-catenin/shh/wnt1/ptc regulation model. (MP4)

S5 Movie Simulation of gap junction communication model. (MP4)

S6 Movie Simulation of classical cuts model. (MP4)

S7 Movie Simulation of comprehensive model of planarian regeneration. (MP4)

S1 File System of equations of inferred regulatory networks. (PDF)
