# Supplementary figures and images for: Inferring Regulatory Networks from Experimental Morphological Phenotypes: A Computational Method Reverse-Engineers Planarian Regeneration
Source: PLoS Comput Biol. 2015 Jun 4;11(6):e1004295. doi: 10.1371/journal.pcbi.1004295 (PMC4456145; doi:10.1371/journal.pcbi.1004295)

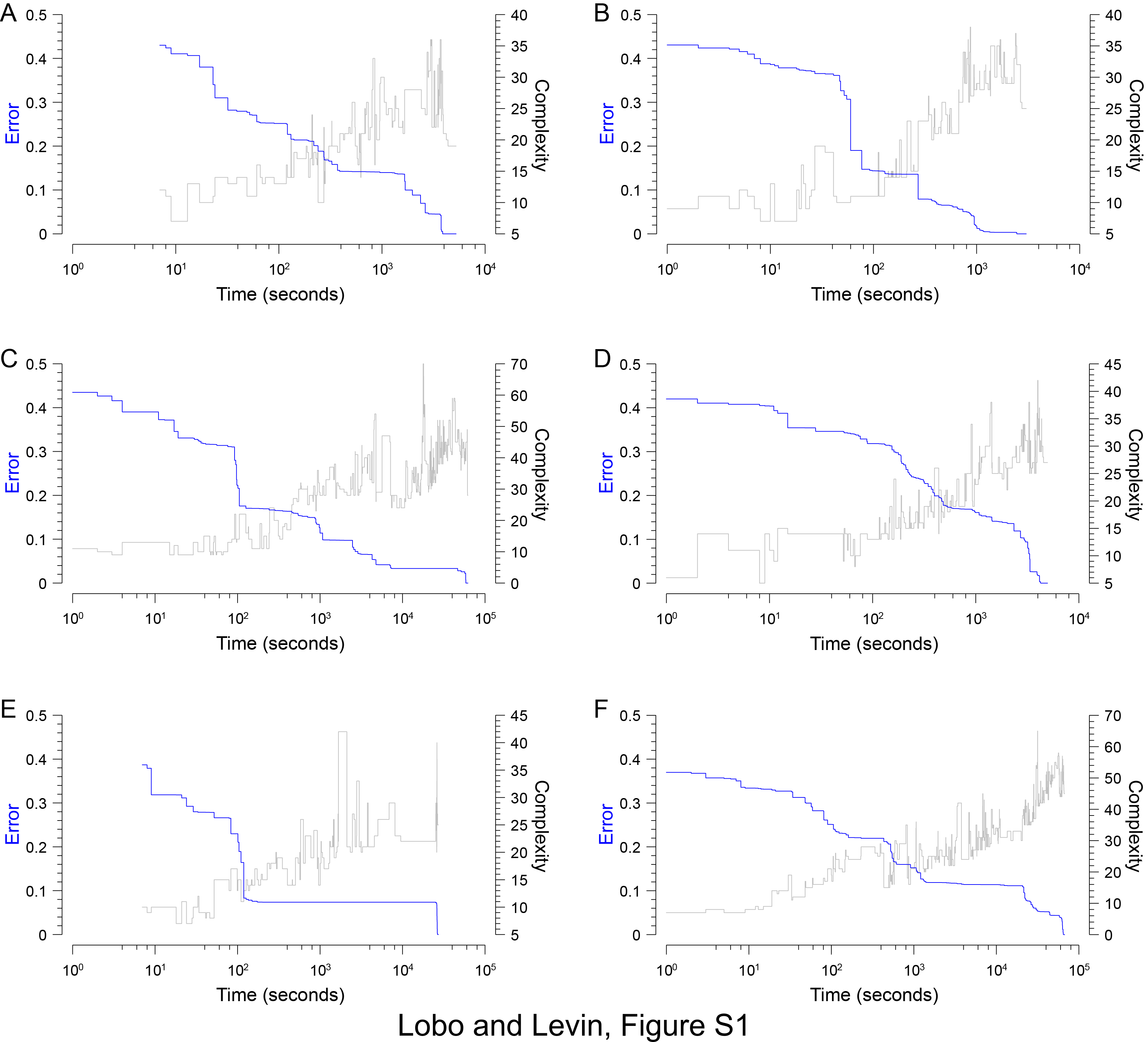

Supplement: S1 Fig — (A-F) Each panel corresponds with the evolutionary search that resulted in the finding of the regulatory networks shown in Fig 3A–3F, respectively. (TIF) [file pcbi.1004295.s001.tif]

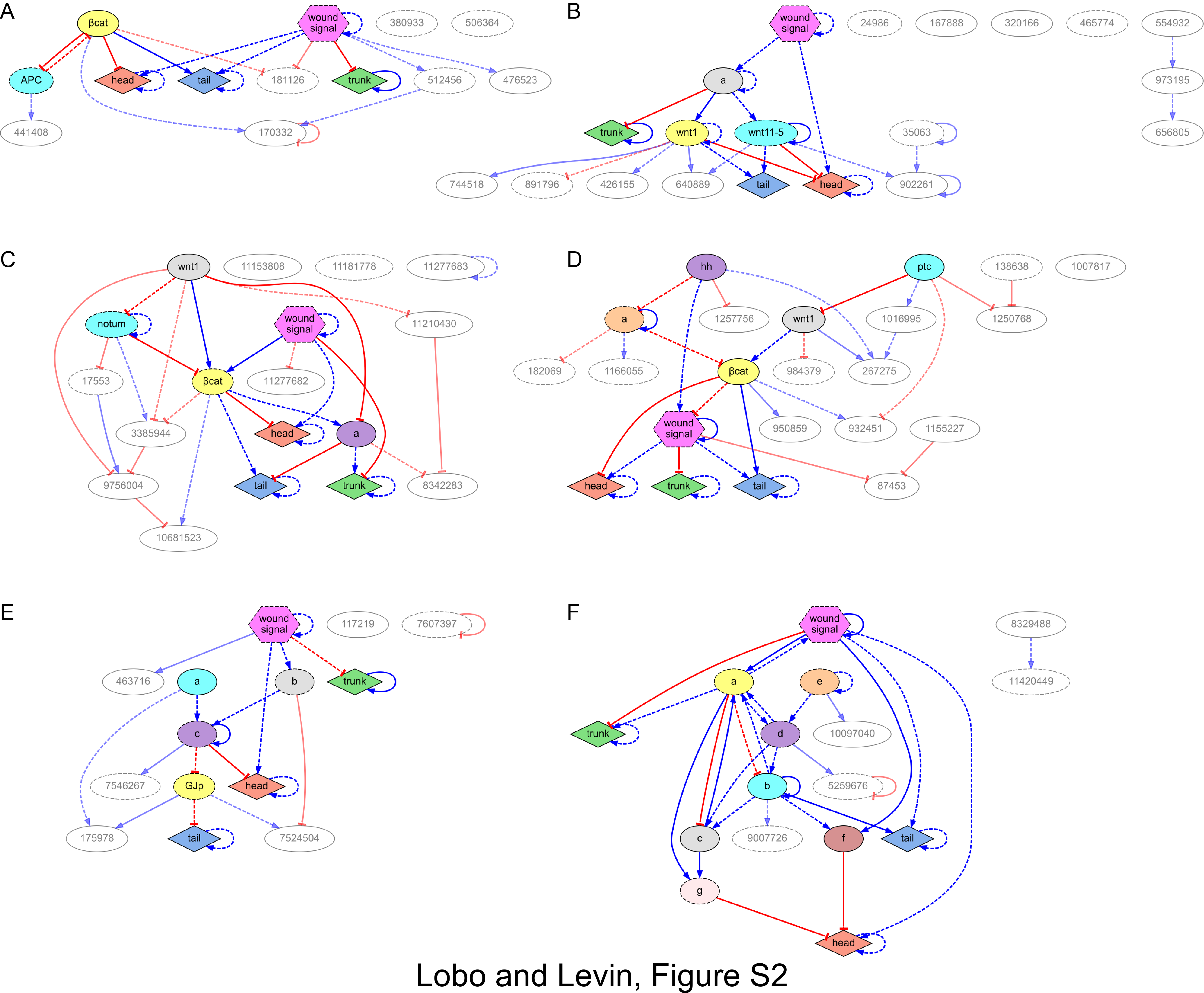

Supplement: S2 Fig — (A-F) Final regulatory networks including known morphological and genetic products, novel products (labeled with letters), and auxiliary products (numbered, light grey) corresponding to the inferred networks shown in Fig 3A–3F, respectively. Both the auxiliary products and novel products are found de novo by the search algorithm; however, in contrast to the novel products, the auxiliary products do not have regulatory interactions affecting directly or indirectly morphological outcomes (head, trunk, and tail products) and hence are not considered part of the final discovered signaling network. (TIF) [file pcbi.1004295.s002.tif]
