# Supplementary material for: Inferring Regulatory Networks from Experimental Morphological Phenotypes: A Computational Method Reverse-Engineers Planarian Regeneration
Source: PLoS Comput Biol. 2015 Jun 4;11(6):e1004295. doi: 10.1371/journal.pcbi.1004295 (PMC4456145; doi:10.1371/journal.pcbi.1004295)
Supplement: S1 File — (PDF) [file pcbi.1004295.s010.pdf]

Inferring Regulatory Networks from Experimental Morphological Phenotypes: a Computational Method Reverse-Engineers Planarian Regeneration

Daniel Lobo and Michael Levin

Supplementary File S1

System of equations of inferred regulatory networks

We present here the complete set of equations, including the kinetics parameters of the regulations, of the inferred networks shown in Figure 3, Figure 6, and Figure 8.

β-catenin/APC regulation model (Fig. 3A)

$$\frac{\partial head}{\partial t} = 0.65 \cdot \min\left(\frac{0.47^{3.4}}{0.47^{3.4} + \beta cat^{3.4}}, \max\left(\frac{head^{1.9}}{0.36^{1.9} + head^{1.9}}, \frac{wound^{1.6}}{0.21^{1.6} + wound^{1.6}}\right)\right) - 0.62 \cdot head$$

$$\frac{\partial trunk}{\partial t} = 0.92 \cdot \min\left(\frac{trunk^{3.9}}{0.61^{3.9} + trunk^{3.9}}, \frac{0.085^{4.3}}{0.085^{4.3} + wound^{4.3}}\right) - 0.83 \cdot trunk$$

$$\frac{\partial tail}{\partial t} = 0.79 \cdot \min\left(\frac{\beta cat^{4}}{0.61^{4} + \beta cat^{4}}, \max\left(\frac{tail^{4}}{0.2^{4} + tail^{4}}, \frac{wound^{1.3}}{0.3^{1.3} + wound^{1.3}}\right)\right) - 0.85 \cdot tail$$

$$\frac{\partial wound}{\partial t} = 0.042 \cdot \frac{wound^{1.4}}{0.32^{1.4} + wound^{1.4}} - 0.19 \cdot wound + 0.93 \cdot \nabla^2 wound$$

$$\frac{\partial \beta cat}{\partial t} = 0.87 \cdot \frac{0.24^{1.8}}{0.24^{1.8} + apc^{1.8}} - 0.33 \cdot \beta cat$$

$$\frac{\partial apc}{\partial t} = 0.99 \cdot \frac{0.56^{4.5}}{0.56^{4.5} + \beta cat^{4.5}} - 0.91 \cdot apc + 0.91 \cdot \nabla^2 apc$$

wnt1/wnt11-5 regulation model (Fig. 3B)

$$\frac{\partial head}{\partial t} = 0.42 \cdot \min\left(\frac{0.056^{4.2}}{0.056^{4.2} + wnt1^{4.2}}, \frac{0.55^{2.7}}{0.55^{2.7} + wnt2^{2.7}}, \max\left(\frac{head^{3.1}}{0.16^{3.1} + head^{3.1}}, \frac{wound^{4.3}}{0.16^{4.3} + wound^{4.3}}\right)\right) - 0.47 \cdot head$$

$$\frac{\partial trunk}{\partial t} = 0.88 \cdot \min\left(\frac{trunk^{2.7}}{0.65^{2.7} + trunk^{2.7}}, \frac{0.89^{1.6}}{0.89^{1.6} + a^{1.6}}\right) - 0.67 \cdot trunk$$

$$\frac{\partial tail}{\partial t} = 0.83 \cdot \max\left(\frac{wnt1^{4.7}}{0.57^{4.7} + wnt1^{4.7}}, \frac{wnt11^{4.1}}{0.75^{4.1} + wnt11^{4.1}}\right) - 0.76 \cdot tail$$

$$\frac{\partial wound}{\partial t} = 0.53 \cdot \frac{wound^{4.3}}{0.57^{4.3} + wound^{4.3}} - 0.26 \cdot wound + 0.94 \cdot \nabla^2 wound$$

$$\frac{\partial wnt1}{\partial t} = 0.82 \cdot \min\left(\frac{a^{2.8}}{0.43^{2.8} + a^{2.8}}, \frac{wnt1^{4.7}}{0.074^{4.7} + wnt1^{4.7}}\right) - 0.46 \cdot wnt1 + 0.093 \cdot \nabla^2 wnt1$$

$$\frac{\partial wnt11}{\partial t} = 0.73 \cdot \min\left(\frac{wnt11^{4.5}}{0.11^{4.5} + wnt11^{4.5}}, \frac{a^{1.1}}{0.22^{1.1} + a^{1.1}}\right) - 0.35 \cdot wnt11 + 0.21 \cdot \nabla^2 wnt11$$

$$\frac{\partial a}{\partial t} = 0.82 \cdot \max\left(\frac{wound^{4}}{0.23^{4} + wound^{4}}, \frac{a^{2.9}}{0.067^{2.9} + a^{2.9}}\right) - 0.83 \cdot a$$

β-catenin/notum/wnt1 regulation model (Fig. 3C)

$$\frac{\partial head}{\partial t} = 0.89 \cdot \min\left(\frac{0.26^{1.2}}{0.26^{1.2} + \beta cat^{1.2}}, \max\left(\frac{head^{3.3}}{0.29^{3.3} + head^{3.3}}, \frac{wound^{4}}{0.11^{4} + wound^{4}}\right)\right) - 0.94 \cdot head$$

$$\frac{\partial trunk}{\partial t} = 0.57 \cdot \min\left(\frac{0.063^{4.7}}{0.063^{4.7} + wound^{4.7}}, \max\left(\frac{trunk^{3.1}}{0.53^{3.1} + trunk^{3.1}}, \frac{a^{1.2}}{0.77^{1.2} + a^{1.2}}\right)\right) - 0.53 \cdot trunk$$

$$\frac{\partial tail}{\partial t} = 0.31 \cdot \min\left(\frac{0.37^{1.3}}{0.37^{1.3} + a^{1.3}}, \max\left(\frac{tail^{2.3}}{0.5^{2.3} + tail^{2.3}}, \frac{\beta cat^{3.4}}{0.88^{3.4} + \beta cat^{3.4}}\right)\right) - 0.28 \cdot tail$$

$$\frac{\partial wound}{\partial t} = 0.41 \cdot \frac{wound^{4}}{0.62^{4} + wound^{4}} - 0.22 \cdot wound + 0.95 \cdot \nabla^{2} wound$$

$$\frac{\partial \beta cat}{\partial t} = 0.72 \cdot \min\left(\frac{wound^{2.1}}{0.054^{2.1} + wound^{2.1}}, \frac{0.017^{4.5}}{0.017^{4.5} + notum^{4.5}}, \frac{wnt1^{1}}{0.26^{1} + wnt1^{1}}\right) - 0.39 \cdot \beta cat + 0.89 \cdot \nabla^{2} \beta cat$$

$$\frac{\partial notum}{\partial t} = 0.23 \cdot \max\left(\frac{notum^{5}}{0.12^{5} + notum^{5}}, \frac{0.073^{2.1}}{0.073^{2.1} + wnt1^{2.1}}\right) - 0.48 \cdot notum + 0.063 \cdot \nabla^{2} notum$$

$$\frac{\partial wnt1}{\partial t} = 0.87 - 0.34 \cdot wnt1$$

$$\frac{\partial a}{\partial t} = 0.41 \cdot \min\left(\frac{0.27^{1.6}}{0.27^{1.6} + wnt1^{1.6}}, \frac{\beta cat^{3.8}}{0.69^{3.8} + \beta cat^{3.8}}\right) - 0.17 \cdot a$$

β-catenin/shh/wnt1/ptc regulation model (Fig. 3D)

$$\frac{\partial head}{\partial t} = 0.86 \cdot \min\left(\frac{0.86^{4.8}}{0.86^{4.8} + \beta cat^{4.8}}, \max\left(\frac{head^{4.1}}{0.27^{4.1} + head^{4.1}}, \frac{wound^{1}}{0.0071^{1} + wound^{1}}\right)\right) - 0.94 \cdot head$$

$$\frac{\partial trunk}{\partial t} = 0.77 \cdot \min\left(\frac{0.0019^{5}}{0.0019^{5} + wound^{5}}, \frac{trunk^{1.4}}{0.028^{1.4} + trunk^{1.4}}\right) - 0.84 \cdot trunk$$

$$\frac{\partial tail}{\partial t} = 0.92 \cdot \min\left(\frac{\beta cat^{3.4}}{0.3^{3.4} + \beta cat^{3.4}}, \max\left(\frac{tail^{2.3}}{0.045^{2.3} + tail^{2.3}}, \frac{wound^{1}}{0.44^{1} + wound^{1}}\right)\right) - 0.9 \cdot tail$$

$$\frac{\partial wound}{\partial t} = 0.16 \cdot \min\left(\frac{wound^{3.6}}{0.096^{3.6} + wound^{3.6}}, \max\left(\frac{0.61^{2.5}}{0.61^{2.5} + \beta cat^{2.5}}, \frac{shh^{4.6}}{0.31^{4.6} + shh^{4.6}}\right)\right) - 0.5 \cdot wound + 0.061 \cdot \nabla^{2} wound$$

$$\frac{\partial \beta cat}{\partial t} = 0.97 \cdot \max\left(\frac{wnt1^{4.1}}{0.27^{4.1} + wnt1^{4.1}}, \frac{0.00086^{4.8}}{0.00086^{4.8} + a^{4.8}}\right) - 0.73 \cdot \beta cat$$

$$\frac{\partial ptc}{\partial t} = 0.56 - 0.86 \cdot ptc$$

$$\frac{\partial wnt1}{\partial t} = 0.52 \cdot \frac{0.11^{3.6}}{0.11^{3.6} + ptc^{3.6}} - 0.19 \cdot wnt1$$

$$\frac{\partial shh}{\partial t} = 0.66 - 0.67 \cdot shh$$

$$\frac{\partial a}{\partial t} = 0.25 \cdot \min\left(\frac{a^{3.5}}{0.1^{3.5} + a^{3.5}}, \frac{0.96^{1}}{0.96^{1} + shh^{1}}\right) - 0.77 \cdot a + 0.31 \cdot \nabla^{2} a$$

Gap junction communication model (Fig. 3E)

$$\frac{\partial head}{\partial t} = 0.8 \cdot \min\left(\frac{0.1^{3.4}}{0.1^{3.4} + c^{3.4}}, \max\left(\frac{head^{2.9}}{0.15^{2.9} + head^{2.9}}, \frac{wound^{2.5}}{0.24^{2.5} + wound^{2.5}}\right)\right) - 0.89 \cdot head$$

$$\frac{\partial trunk}{\partial t} = 0.45 \cdot \min\left(\frac{trunk^{3.8}}{0.34^{3.8} + trunk^{3.8}}, \frac{0.063^{4.9}}{0.063^{4.9} + wound^{4.9}}\right) - 0.49 \cdot trunk$$

$$\frac{\partial tail}{\partial t} = 0.99 \cdot \max\left(\frac{tail^{4.4}}{0.18^{4.4} + tail^{4.4}}, \frac{0.2^{2.4}}{0.2^{2.4} + GJp^{2.4}}\right) - 0.95 \cdot tail$$

$$\frac{\partial wound}{\partial t} = 0.9 \cdot \frac{wound^{1.9}}{0.72^{1.9} + wound^{1.9}} - 0.46 \cdot wound + 0.87 \cdot \nabla^2 wound$$

$$\frac{\partial GJp}{\partial t} = 0.48 \cdot \frac{0.55^{3.8}}{0.55^{3.8} + c^{3.8}} - 0.57 \cdot GJp + 0.25 \cdot \nabla^2 GJp$$

$$\frac{\partial a}{\partial t} = 0.11 - 0.92 \cdot a$$

$$\frac{\partial b}{\partial t} = 0.83 \cdot \frac{wound^{4.1}}{0.018^{4.1} + wound^{4.1}} - 0.18 \cdot b + 1 \cdot \nabla^2 b$$

$$\frac{\partial c}{\partial t} = 0.59 \cdot \min\left(\frac{c^{2.4}}{0.57^{2.4} + c^{2.4}}, \max\left(\frac{a^{4.3}}{0.13^{4.3} + a^{4.3}}, \frac{b^{2.5}}{0.24^{2.5} + b^{2.5}}\right)\right) - 0.37 \cdot c + 0.19 \cdot \nabla^2 c$$

Classical cuts model (Fig. 3F)

$$\frac{\partial head}{\partial t} = 0.41 \cdot \min\left(\frac{0.23^{2.6}}{0.23^{2.6} + f^{2.6}}, \frac{0.87^{2.9}}{0.87^{2.9} + g^{2.9}}, \max\left(\frac{head^{3}}{0.53^{3} + head^{3}}, \frac{wound^{3.8}}{0.0071^{3.8} + wound^{3.8}}\right)\right) - 0.37 \cdot head$$

$$\frac{\partial trunk}{\partial t} = 0.5 \cdot \min\left(\frac{0.0047^{4.9}}{0.0047^{4.9} + wound^{4.9}}, \max\left(\frac{trunk^{2.6}}{0.3^{2.6} + trunk^{2.6}}, \frac{a^{1.5}}{0.58^{1.5} + a^{1.5}}\right)\right) - 0.51 \cdot trunk$$

$$\frac{\partial tail}{\partial t} = 0.66 \cdot \min\left(\frac{b^{4}}{0.047^{4} + b^{4}}, \max\left(\frac{tail^{2.7}}{0.17^{2.7} + tail^{2.7}}, \frac{wound^{1}}{0.064^{1} + wound^{1}}\right)\right) - 0.62 \cdot tail$$

$$\frac{\partial wound}{\partial t} = 0.35 \cdot \min\left(\frac{wound^{4.9}}{0.18^{4.9} + wound^{4.9}}, \frac{a^{3.6}}{0.08^{3.6} + a^{3.6}}\right) - 0.85 \cdot wound + 0.49 \cdot \nabla^2 wound$$

$$\frac{\partial a}{\partial t} = 0.98 \cdot \min\left(\frac{wound^{1.7}}{0.00041^{1.7} + wound^{1.7}}, \frac{c^{3.7}}{0.61^{3.7} + c^{3.7}}, \max\left(\frac{b^{4.9}}{0.67^{4.9} + b^{4.9}}, \frac{d^{3.3}}{0.62^{3.3} + d^{3.3}}\right)\right) - 0.12 \cdot a + 0.43 \cdot \nabla^2 a$$

$$\frac{\partial b}{\partial t} = 0.61 \cdot \min\left(\frac{b^{3.4}}{0.34^{3.4} + b^{3.4}}, \max\left(\frac{0.097^{5}}{0.097^{5} + a^{5}}, \frac{d^{1.7}}{0.96^{1.7} + d^{1.7}}\right)\right) - 0.42 \cdot b$$

$$\frac{\partial c}{\partial t} = 0.58 \cdot \min\left(\frac{0.4^{4.9}}{0.4^{4.9} + a^{4.9}}, \max\left(\frac{b^{2.9}}{0.7^{2.9} + b^{2.9}}, \frac{d^{4.4}}{0.54^{4.4} + d^{4.4}}\right)\right) - 0.14 \cdot c$$

$$\frac{\partial d}{\partial t} = 0.51 \cdot \max\left(\frac{a^{3.5}}{0.93^{3.5} + a^{3.5}}, \frac{e^{1.8}}{0.0026^{1.8} + e^{1.8}}\right) - 0.33 \cdot d + 0.96 \cdot \nabla^2 d$$

$$\frac{\partial e}{\partial t} = 0.74 \cdot \frac{e^{3.4}}{0.99^{3.4} + e^{3.4}} - 0.3 \cdot e + 0.076 \cdot \nabla^2 e$$

$$\frac{\partial f}{\partial t} = 0.87 \cdot \min\left(\frac{wound^{3.3}}{0.0067^{3.3} + wound^{3.3}}, \frac{b^{1.6}}{0.13^{1.6} + b^{1.6}}\right) - 0.63 \cdot f$$

$$\frac{\partial g}{\partial t} = 0.7 \cdot \min\left(\frac{a^{3.1}}{0.51^{3.1} + a^{3.1}}, \frac{c^{3.4}}{0.9^{3.4} + c^{3.4}}\right) - 0.12 \cdot g + 0.73 \cdot \nabla^2 g$$

Comprehensive model of planarian regeneration (Fig. 8)

$$\frac{\partial head}{\partial t} = 0.87 \cdot \min\left(\frac{0.18^{4.6}}{0.18^{4.6} + \beta cat^{4.6}}, \frac{a^{4.3}}{0.095^{4.3} + a^{4.3}}, \max\left(\frac{head^{3.1}}{0.11^{3.1} + head^{3.1}}, \frac{wound^{4.2}}{0.17^{4.2} + wound^{4.2}}\right)\right) - 0.97 \cdot head$$

$$\frac{\partial trunk}{\partial t} = 0.92 \cdot \min\left(\frac{0.073^{5}}{0.073^{5} + wound^{5}}, \max\left(\frac{trunk^{3.3}}{0.38^{3.3} + trunk^{3.3}}, \frac{b^{3.1}}{0.6^{3.1} + b^{3.1}}\right)\right) - 0.96 \cdot trunk$$

$$\frac{\partial tail}{\partial t} = 0.92 \cdot \min\left(\frac{shh^{1.5}}{0.017^{1.5} + shh^{1.5}}, \max\left(\frac{tail^{2.8}}{0.51^{2.8} + tail^{2.8}}, \frac{0.14^{1.7}}{0.14^{1.7} + a^{1.7}}\right)\right) - 0.85 \cdot tail$$

$$\frac{\partial wound}{\partial t} = 0.93 \cdot \frac{wnt11^{5}}{0.7^{5} + wnt11^{5}} - 0.065 \cdot wound + 0.86 \cdot \nabla^2 wound$$

$$\frac{\partial GJp}{\partial t} = 0.85 \cdot \frac{notum^{3.2}}{0.17^{3.2} + notum^{3.2}} - 0.66 \cdot GJp + 0.98 \cdot \nabla^2 GJp$$

$$\frac{\partial \beta cat}{\partial t} = 0.64 \cdot \min\left(\frac{wound^{1.2}}{0.18^{1.2} + wound^{1.2}}, \frac{wnt1^{1.8}}{0.44^{1.8} + wnt1^{1.8}}, \frac{wnt11^{1.2}}{0.14^{1.2} + wnt11^{1.2}}, \max\left(\frac{0.18^{3.8}}{0.18^{3.8} + apc^{3.8}}, \frac{0.17^{2.6}}{0.17^{2.6} + ptc^{2.6}}\right)\right) - 0.49 \cdot \beta cat + 0.11 \cdot \nabla^2 \beta cat$$

$$\frac{\partial notum}{\partial t} = 0.65 \cdot \frac{apc^{3.6}}{0.21^{3.6} + apc^{3.6}} - 0.81 \cdot notum$$

$$\frac{\partial wnt1}{\partial t} = 0.34 \cdot \frac{0.27^{1.4}}{0.27^{1.4} + notum^{1.4}} - 0.16 \cdot wnt1$$

$$\frac{\partial wnt11}{\partial t} = 0.053 - 0.76 \cdot wnt11$$

$$\frac{\partial apc}{\partial t} = 0.46 \cdot \min\left(\frac{0.21^{1.5}}{0.21^{1.5} + wnt11^{1.5}}, \max\left(\frac{wound^{2.5}}{0.37^{2.5} + wound^{2.5}}, \frac{notum^{2.7}}{0.35^{2.7} + notum^{2.7}}\right)\right) - 0.93 \cdot apc + 0.86 \cdot \nabla^2 apc$$

$$\frac{\partial shh}{\partial t} = 0.98 \cdot \max\left(\frac{GJp^{2.9}}{0.013^{2.9} + GJp^{2.9}}, \frac{wnt1^{4.2}}{1^{4.2} + wnt1^{4.2}}\right) - 0.68 \cdot shh$$

$$\frac{\partial ptc}{\partial t} = 0.94 - 0.64 \cdot ptc$$

$$\frac{\partial a}{\partial t} = 0.49 \cdot \min\left(\frac{0.26^{4.7}}{0.26^{4.7} + \beta cat^{4.7}}, \max\left(\frac{GJp^{4.8}}{0.35^{4.8} + GJp^{4.8}}, \frac{0.94^{4.9}}{0.94^{4.9} + wnt1^{4.9}}, \frac{wnt11^{4.8}}{0.0015^{4.8} + wnt11^{4.8}}\right)\right) - 0.71 \cdot a$$

$$\frac{\partial b}{\partial t} = 0.98 \cdot \min\left(\frac{\beta cat^{4.9}}{0.24^{4.9} + \beta cat^{4.9}}, \frac{0.067^{4.4}}{0.067^{4.4} + shh^{4.4}}\right) - 0.45 \cdot b$$

Candidate system of equations during a search

Here we present the complete set of equations, including the kinetics parameters of the regulations, of the candidate networks shown in Figure 6.

Generation 0

$$\frac{\partial head}{\partial t} = 0.13 \cdot \min\left(\frac{0.38^{4}}{0.38^{4} + notum^{4}}, \frac{head^{4}}{0.95^{4} + head^{4}}\right) - 0.96 \cdot head$$

$$\frac{\partial trunk}{\partial t} = 0.48 - 0.54 \cdot trunk$$

$$\frac{\partial tail}{\partial t} = 0.73 \cdot \min\left(\frac{\beta cat^{4}}{0.6^{4} + \beta cat^{4}}, \frac{tail^{2.9}}{0.23^{2.9} + tail^{2.9}}\right) - 0.76 \cdot tail$$

$$\frac{\partial wound}{\partial t} = 0.82 - 0.55 \cdot wound + 0.64 \cdot \nabla^2 wound$$

$$\frac{\partial \beta cat}{\partial t} = 0.47 - 0.12 \cdot \beta cat$$

$$\frac{\partial notum}{\partial t} = 0.79 - 0.67 \cdot notum$$

Generation 50

$$\frac{\partial head}{\partial t} = 0.93 \cdot \frac{head^{2.4}}{0.38^{2.4} + head^{2.4}} - 0.82 \cdot head$$
$$\frac{\partial trunk}{\partial t} = 0.57 \cdot \frac{trunk^{2.9}}{0.53^{2.9} + trunk^{2.9}} - 0.53 \cdot trunk$$
$$\frac{\partial tail}{\partial t} = 0.64 \cdot \frac{tail^{2.9}}{0.29^{2.9} + tail^{2.9}} - 0.65 \cdot tail + 0.01 \cdot \nabla^2 tail$$
$$\frac{\partial wound}{\partial t} = 0.91 - 0.95 \cdot wound$$

Generation 100

$$\frac{\partial head}{\partial t} = 0.54 \cdot \frac{0.79^{1.3}}{0.79^{1.3} + \beta cat^{1.3}} - 0.61 \cdot head$$
$$\frac{\partial trunk}{\partial t} = 0.91 \cdot \frac{\beta cat^{1.2}}{0.86^{1.2} + \beta cat^{1.2}} - 0.9 \cdot trunk$$
$$\frac{\partial tail}{\partial t} = 1 \cdot \frac{tail^{2.6}}{0.26^{2.6} + tail^{2.6}} - 0.97 \cdot tail$$
$$\frac{\partial wound}{\partial t} = 0.19 - 0.29 \cdot wound$$
$$\frac{\partial \beta cat}{\partial t} = 0.55 \cdot \frac{\beta cat^{3.4}}{0.95^{3.4} + \beta cat^{3.4}} - 0.1 \cdot \beta cat$$

Generation 200

$$\frac{\partial head}{\partial t} = 0.84 \cdot \max\left(\frac{head^{3.3}}{0.29^{3.3} + head^{3.3}}, \frac{wound^{4.9}}{0.11^{4.9} + wound^{4.9}}\right) - 0.85 \cdot head$$
$$\frac{\partial trunk}{\partial t} = 0.73 \cdot \min\left(\frac{trunk^{4.2}}{0.46^{4.2} + trunk^{4.2}}, \frac{0.04^{3.1}}{0.04^{3.1} + wound^{3.1}}\right) - 0.73 \cdot trunk$$
$$\frac{\partial tail}{\partial t} = 0.16 \cdot \frac{tail^{1.6}}{0.92^{1.6} + tail^{1.6}} - 0.086 \cdot tail$$
$$\frac{\partial wound}{\partial t} = 0.23 \cdot \min\left(\frac{\beta cat^{3.6}}{0.47^{3.6} + \beta cat^{3.6}}, \frac{wound^{4.2}}{0.16^{4.2} + wound^{4.2}}\right) - 0.46 \cdot wound + 0.47 \cdot \nabla^2 wound$$
$$\frac{\partial \beta cat}{\partial t} = 0.14 - 0.48 \cdot \beta cat$$

Generation 1000

$$\frac{\partial head}{\partial t} = 0.17 \cdot \min\left(\frac{0.17^{1.5}}{0.17^{1.5} + wnt1^{1.5}}, \frac{0.28^{1.6}}{0.28^{1.6} + a^{1.6}}, \max\left(\frac{head^{1.9}}{0.031^{1.9} + head^{1.9}}, \frac{wound^{1.9}}{0.31^{1.9} + wound^{1.9}}\right)\right) - 0.15 \cdot head$$
$$\frac{\partial trunk}{\partial t} = 0.37 \cdot \min\left(\frac{0.0038^{2.1}}{0.0038^{2.1} + wound^{2.1}}, \frac{trunk^{4.9}}{0.2^{4.9} + trunk^{4.9}}\right) - 0.4 \cdot trunk$$
$$\frac{\partial tail}{\partial t} = 0.66 \cdot \max\left(\frac{tail^{2.9}}{0.33^{2.9} + tail^{2.9}}, \frac{wnt1^{3}}{0.79^{3} + wnt1^{3}}\right) - 0.65 \cdot tail$$
$$\frac{\partial wound}{\partial t} = 0.24 \cdot \frac{wound^{3.6}}{0.59^{3.6} + wound^{3.6}} - 0.2 \cdot wound + 0.16 \cdot \nabla^2 wound$$
$$\frac{\partial \beta cat}{\partial t} = 0.93 \cdot \frac{0.038^{3}}{0.038^{3} + notum^{3}} - 0.46 \cdot \beta cat + 0.36 \cdot \nabla^2 \beta cat$$
$$\frac{\partial notum}{\partial t} = 0.8 \cdot \frac{\beta cat^{4.9}}{0.2^{4.9} + \beta cat^{4.9}} - 0.97 \cdot notum$$
$$\frac{\partial wnt1}{\partial t} = 0.51 \cdot \frac{\beta cat^{3.3}}{0.56^{3.3} + \beta cat^{3.3}} - 0.32 \cdot wnt1$$
$$\frac{\partial a}{\partial t} = 0.16 \cdot \frac{0.27^{1.1}}{0.27^{1.1} + a^{1.1}} - 0.92 \cdot a$$

Generation 4000

$$\frac{\partial head}{\partial t} = 0.88 \cdot \min\left(\frac{0.21^{1}}{0.21^{1} + \beta cat^{1}}, \max\left(\frac{head^{3.2}}{0.29^{3.2} + head^{3.2}}, \frac{wound^{3.2}}{0.11^{3.2} + wound^{3.2}}\right)\right) - 0.95 \cdot head$$

$$\frac{\partial trunk}{\partial t} = 0.73 \cdot \min\left(\frac{trunk^{4.7}}{0.37^{4.7} + trunk^{4.7}}, \frac{0.036^{4.7}}{0.036^{4.7} + wound^{4.7}}\right) - 0.73 \cdot trunk + 0.069 \cdot \nabla^2 trunk$$

$$\frac{\partial tail}{\partial t} = 0.31 \cdot \max\left(\frac{tail^{2.9}}{0.49^{2.9} + tail^{2.9}}, \frac{\beta cat^{2.8}}{0.86^{2.8} + \beta cat^{2.8}}\right) - 0.28 \cdot tail$$

$$\frac{\partial wound}{\partial t} = 0.41 \cdot \frac{wound^{4.3}}{0.62^{4.3} + wound^{4.3}} - 0.21 \cdot wound + 0.68 \cdot \nabla^2 wound$$

$$\frac{\partial \beta cat}{\partial t} = 0.44 \cdot \min\left(\frac{0.022^{1.8}}{0.022^{1.8} + notum^{1.8}}, \max\left(\frac{wound^{4.3}}{0.048^{4.3} + wound^{4.3}}, \frac{wnt1^{4.4}}{0.62^{4.4} + wnt1^{4.4}}\right)\right) - 0.45 \cdot \beta cat + 0.57 \cdot \nabla^2 \beta cat$$

$$\frac{\partial notum}{\partial t} = 0.23 \cdot \frac{notum^{4.9}}{0.12^{4.9} + notum^{4.9}} - 0.49 \cdot notum + 0.063 \cdot \nabla^2 notum$$

$$\frac{\partial wnt1}{\partial t} = 0.71 \cdot \frac{wound^{3}}{0.042^{3} + wound^{3}} - 0.3 \cdot wnt1 + 0.66 \cdot \nabla^2 wnt1$$

Generation 10000

$$\frac{\partial head}{\partial t} = 0.88 \cdot \min\left(\frac{0.26^{1.2}}{0.26^{1.2} + \beta cat^{1.2}}, \max\left(\frac{head^{3.3}}{0.29^{3.3} + head^{3.3}}, \frac{wound^{3.4}}{0.11^{3.4} + wound^{3.4}}\right)\right) - 0.95 \cdot head$$

$$\frac{\partial trunk}{\partial t} = 0.8 \cdot \min\left(\frac{0.036^{1.6}}{0.036^{1.6} + wound^{1.6}}, \max\left(\frac{trunk^{4.9}}{0.4^{4.9} + trunk^{4.9}}, \frac{a^{2.4}}{0.84^{2.4} + a^{2.4}}\right)\right) - 0.72 \cdot trunk$$

$$\frac{\partial tail}{\partial t} = 0.31 \cdot \max\left(\frac{tail^{2.3}}{0.5^{2.3} + tail^{2.3}}, \frac{\beta cat^{2.5}}{0.88^{2.5} + \beta cat^{2.5}}, \frac{0.096^{1.5}}{0.096^{1.5} + b^{1.5}}\right) - 0.28 \cdot tail$$

$$\frac{\partial wound}{\partial t} = 0.41 \cdot \frac{wound^{4.3}}{0.62^{4.3} + wound^{4.3}} - 0.21 \cdot wound + 0.9 \cdot \nabla^2 wound$$

$$\frac{\partial \beta cat}{\partial t} = 0.72 \cdot \min\left(\frac{0.017^{2.2}}{0.017^{2.2} + notum^{2.2}}, \frac{wnt1^{1.1}}{0.25^{1.1} + wnt1^{1.1}}, \frac{wound^{2.1}}{0.054^{2.1} + wound^{2.1}}\right) - 0.39 \cdot \beta cat + 0.89 \cdot \nabla^2 \beta cat$$

$$\frac{\partial notum}{\partial t} = 0.23 \cdot \frac{notum^{4.7}}{0.12^{4.7} + notum^{4.7}} - 0.5 \cdot notum + 0.063 \cdot \nabla^2 notum$$

$$\frac{\partial wnt1}{\partial t} = 0.85 - 0.36 \cdot wnt1$$

$$\frac{\partial a}{\partial t} = 0.27 - 0.84 \cdot a$$

$$\frac{\partial b}{\partial t} = 0.94 \cdot \frac{0.22^{4.2}}{0.22^{4.2} + wound^{4.2}} - 0.11 \cdot b$$

Generation 26722

$$\frac{\partial head}{\partial t} = 0.89 \cdot \min\left(\frac{0.26^{1.2}}{0.26^{1.2} + \beta cat^{1.2}}, \max\left(\frac{head^{3.3}}{0.29^{3.3} + head^{3.3}}, \frac{wound^{4}}{0.11^{4} + wound^{4}}\right)\right) - 0.94 \cdot head$$

$$\frac{\partial trunk}{\partial t} = 0.57 \cdot \min\left(\frac{0.063^{4.7}}{0.063^{4.7} + wound^{4.7}}, \max\left(\frac{trunk^{3.1}}{0.53^{3.1} + trunk^{3.1}}, \frac{a^{1.2}}{0.77^{1.2} + a^{1.2}}\right)\right) - 0.53 \cdot trunk$$

$$\frac{\partial tail}{\partial t} = 0.31 \cdot \min\left(\frac{0.37^{1.3}}{0.37^{1.3} + a^{1.3}}, \max\left(\frac{tail^{2.3}}{0.5^{2.3} + tail^{2.3}}, \frac{\beta cat^{3.4}}{0.88^{3.4} + \beta cat^{3.4}}\right)\right) - 0.28 \cdot tail$$

$$\frac{\partial wound}{\partial t} = 0.41 \cdot \frac{wound^{4}}{0.62^{4} + wound^{4}} - 0.22 \cdot wound + 0.95 \cdot \nabla^2 wound$$

$$\frac{\partial \beta cat}{\partial t} = 0.72 \cdot \min\left(\frac{wound^{2.1}}{0.054^{2.1} + wound^{2.1}}, \frac{0.017^{4.5}}{0.017^{4.5} + notum^{4.5}}, \frac{wnt1^{1}}{0.26^{1} + wnt1^{1}}\right) - 0.39 \cdot \beta cat + 0.89 \cdot \nabla^2 \beta cat$$

$$\frac{\partial notum}{\partial t} = 0.23 \cdot \max\left(\frac{notum^{5}}{0.12^{5} + notum^{5}}, \frac{0.073^{2.1}}{0.073^{2.1} + wnt1^{2.1}}\right) - 0.48 \cdot notum + 0.063 \cdot \nabla^2 notum$$

$$\frac{\partial wnt1}{\partial t} = 0.87 - 0.34 \cdot wnt1$$

$$\frac{\partial a}{\partial t} = 0.41 \cdot \min\left(\frac{0.27^{1.6}}{0.27^{1.6} + wnt1^{1.6}}, \frac{\beta cat^{3.8}}{0.69^{3.8} + \beta cat^{3.8}}\right) - 0.17 \cdot a$$
